# Soil Decomposer Can Regulate the Legacy Effect of Photodegradation on Forest Marcescent Litter Decomposition, but Emerging Microplastics Disrupt This

**DOI:** 10.1002/ece3.70918

**Published:** 2025-01-28

**Authors:** Kai Tian, Xin Wang, Rumeng Ye, Yingqi Wang, Zhicheng Chen, Xiaojing Liu, Wenxia Wang, Lunguang Yao

**Affiliations:** ^1^ Henan Field Observation and Research Station of Headwork Wetland Ecosystem of the Central Route of South‐to‐North Water Diversion Project, School of Life Sciences and Agricultural Engineering Nanyang Normal University Nanyang China; ^2^ Henan International Joint Laboratory of Watershed Ecological Security in the Water Source Area of the Middle Route of South‐to‐North Water Diversion Project Nanyang China; ^3^ Key Laboratory of Forest Ecology and Environment of National Forestry and Grassland Administration Ecology and Nature Conservation Institute, Chinese Academy of Forestry Beijing China; ^4^ Baotianman National Nature Reserve Nanyang China

**Keywords:** biogeochemical process, biotic decomposition, litter quality, marcescence, soil contamination, ultraviolet radiation

## Abstract

Photodegradation—photochemical mineralization of standing litters—often exerts a legacy effect aiding biodegradation in soil (PLE), which is overlooked in deciduous forests containing marcescent leaves. Meanwhile, increasing anthropogenic microplastics have deposited in forests, how they would affect the PLE on subsequent litter bio‐decomposition is currently unknown. Here, we employed an ultraviolet‐accelerated aging chamber to replicate the abiotic photodegradation process of a naturally marcescent tree, *Lindera glauca*, then manipulated mesocosm bio‐incubations to quantify how decomposers (microbial alone or with soil animals) and microplastic contamination would interactively affect the PLE. We found abiotic photodegradation significantly decreased litter lignin content before and after the bio‐incubation. During an early phase decomposition, lignin lost greatly and displayed a crucial role in determining the ways that soil animal and photodegradation affect the bio‐decomposition. Microbial decomposer alone led to a positive PLE universally. Soil animals depressed microbial biomass and inhibited the microbial‐mediated PLE in unpolluted mesocosms but intensified the PLE in contaminated soils. We conclude that decomposer interactions can attenuate PLE, but microplastics will disrupt the established equilibrium, making contaminated soil more susceptible to photodegradation‐induced litter chemical changes. This promotes integration of radiation and emerging pollution to further our understanding of biogeochemical cycle in forest ecology.

## Graphical abstract

Photodegradation—photochemical mineralization of standing litters—often exerts a legacy effect aiding biodegradation in soil (PLE), which is overlooked in deciduous forests containing marcescent leaves. Meanwhile, increasing anthropogenic microplastics have deposited in forests, how they would affect the PLE on subsequent litter bio‐decomposition is currently unknown. Here, we employed an ultraviolet‐accelerated aging chamber to replicate the abiotic photodegradation process of a naturally marcescent tree, Lindera glauca, then manipulated mesocosm bio‐incubations to quantify how decomposers (microbial alone or with soil animals) and microplastic contamination would interactively affect the PLE. We found abiotic photodegradation significantly decreased litter lignin content before and after the bio‐incubation. During an early phase decomposition, lignin lost greatly and displayed a crucial role in determining the ways that soil animal and photodegradation affect the bio‐decomposition. Microbial decomposer alone led to a positive PLE universally. Soil animals depressed microbial biomass and inhibited the microbial‐mediated PLE in unpolluted mesocosms but intensified the PLE in contaminated soils. We conclude that decomposer interactions can attenuate PLE, but microplastics will disrupt the established equilibrium, making contaminated soil more susceptible to photodegradation‐induced litter chemical changes. This arouses integration of radiation and emerging pollution to further our understanding of biogeochemical cycles in forest ecology.

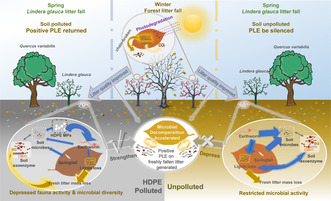

## 1 Introduction

In terrestrial ecosystems, up to 90% of the photosynthetically fixed plant biomass becomes senescent tissues shed into the soil annually, and a considerable amount of these litters are ultimately decomposed (Bardgett 2005; Cebrian 1999). Litter decomposition is the core process of the carbon budget and nutrient cycle in ecosystems (Attiwill and Adams 1993; Hättenschwiler, Tiunov, and Scheu 2005). It is typically thought of as a biological enzymatic process mainly controlled by climate (principally temperature and precipitation), litter quality, and decomposers (Swift, Heal, and Anderson 1979). As an important climatic factor, solar radiation, especially blue light and ultraviolet (UV) radiation, can break down litter through direct photochemical mineralization of complex macromolecules and release smaller organic compounds, a phenomenon known as photodegradation (Austin and Ballaré 2010; Austin, Méndez, and Ballaré 2016). Meanwhile, litter that undergoes solar radiation in the air may increase its chemical quality (e.g., degraded recalcitrant lignin and promoted biodegradability) and generate a legacy effect of photodegradation (PLE), thereby facilitating subsequent bio‐decomposition when the litter encounters the soil (Austin, Méndez, and Ballaré 2016; Lin et al. 2018; Marinho et al. 2020).

Previous research has extensively investigated photodegradation in semi‐arid steppe ecosystems, where solar radiation can directly contribute to up to 40% of the mass loss of litters (Austin and Vivanco 2006). However, the consideration of photodegradation in biogeochemical models for forest ecosystems has been limited, primarily due to the reduced solar radiation reaching the forest litter layers caused by the dense tree canopy. Nevertheless, the photodegradation and its influence on subsequent litter decomposition in soil are critical issues that should not be overlooked in forests. For instance, numerous forest gaps experience ample solar irradiation, thereby greatly contributing to litter decomposition through photodegradation (Wang et al. 2021). Additionally, the seasonal canopy phenology in deciduous leads to heightened light transmittance onto the forest floor during the off‐growing season, a factor that is often disregarded. Other than litters that have already fallen onto soil, the senescent litters that remain marcescent on branches or are intercepted by the understory can initiate a temporary process of photodegradation and weathering, preceding subsequent soil decomposition (Angst et al. 2017; Lin et al. 2018; Jiang et al. 2023). *Lindera glauca*, a unique deciduous tree commonly referred to as “fake death wood”, exhibits a distinctive behavior in which its senescent leaf litters persistently remain attached to the canopy branches rather than falling to the soil during the dormant autumn and winter seasons. These leaf litters only shed after new leaves have spread in the subsequent spring. This characteristic renders the leaf litters susceptible to solar radiation, making them an ideal material for investigating the specific photodegradation and its potential impacts on biogeochemical cycling within forest soil. A recent study conducted by Jiang et al. (2023) confirmed that the marcescent process can enhance subsequent litter decomposition rates of *L. glauca* in soil (Jiang et al. 2023). However, how the abiotic photodegradation during this process could affect litter decomposition and consequent C balance remains unclear.

Especially, while the absence of foliage during the non‐growing season allows for adequate irradiance penetration in forests, it also poses an increased risk of soil exposure to diverse emerging contaminants such as microplastics (MPs, particles or fragments with a diameter less than 5 mm). MPs can accumulate in forest soils through atmospheric deposition, wind dispersal, and anthropogenic activities. In general, the significance of forest soils as a hidden hub of global pollution has been largely disregarded in previous studies (Weber, Rillig, and Bigalke 2023). Recent studies have revealed that MPs are primarily captured by canopies and subsequently transported to the soil via litter fall, rainfall, and runoff, ultimately becoming incorporated into forest soil (Büks and Kaupenjohann 2020; Rillig and Lehmann 2020). This emerging contaminant not only alters the physiochemical composition of the soil but also exerts significant effects on decomposers. The presence of MPs can impede the growth, metabolism, and community structure of soil organisms, as well as inflict damage on the digestive systems of soil biota, thereby negatively impacting soil decomposer activity and nutrient cycling (Cheng et al. 2021). Taking high‐density polyethylene microplastics (HDPE‐MPs) as an example, it can significantly decrease the earthworm biomass (Boots, Russell, and Green 2019). Integrating microplastics into the decomposition system will further our understanding of soil biogeochemical cycles (Rillig and Lehmann 2020; Zhang et al. 2021). Investigating the impact of MPs on PLE, particularly in relation to certain decomposers (microbes, animals, and their interactions), would contribute to our comprehension of the underlying causes of the ecological risk posed by MPs to deciduous forests.

Furthermore, previous studies examined the impacts of photodegradation on litter decomposition mainly by implementing supplementary UV radiation treatments or employing specific filtrations to monitor irradiance in laboratory or field settings (Wang et al. 2021). However, it is important to note that field investigation on litter photodegradation encompasses additional significant processes, including the alternating dry‐wet pulses and bio‐decomposition by the phyllosphere microorganisms (Lin et al. 2018; Fanin et al. 2021). Meanwhile, in the process of photodegradation of litter, lignin is characterized by its abundant phenyl units, which have strong absorptions of shortwave solar radiations. Consequently, lignin is recognized not only for its resistance to biodegradation but also for its sensitivity to light. Notably, natural photochemical weathering of marcescent litter not only encompasses the abiotic aging (photodegradation) but also biodegradation in the phyllosphere during the dry‐wet cycles (García‐Palacios et al. 2016). Therefore, the distinguished contribution of purely abiotic irradiation (litter under laboratory‐controlled experiments without any interference from external environmental factors) on litter decomposition and nutrient release has not been specially resolved. To address this, we employed a UV‐accelerated weathering chamber to simulate the exclusive abiotic photodegradation of marcescent *L. glauca* litters while eliminating any potential biotic interferences. Then, a series of mesocosms was conducted to investigate the interconnected effects of pre‐photodegradation (causality of initial litter quality changes), soil animals (in the presence or absence, denoting divergent decomposer structures), and HDPE‐MPs (with or without MPs pollution) on *L. glauca* litter decomposition.

Here, we identified litter chemical changes and decomposer activities regarding the biodecomposition. PLEs were evaluated by comparing chemical loss rates between litters that were pre‐irradiated or not. Additionally, we developed a structural equation model (SEM) to examine key causalities concerning decomposition rate and to synthesize the direct and indirect explanatory effects of abiotic photodegradation and soil animals on the variations of litter mass loss. Specifically, SEM was conducted separately in two contrasting environments, which were distinguished by whether they were polluted by HDPE‐MPs, so as to examine the potential alterations caused by MPs. We hypothesized that (1) The abiotic aging process (UV radiation) could decrease litter lignin content through photodegradation, thereby indirectly affecting the decomposition in soil and triggering a positive PLE; (2) Soil animals would accelerate litter decomposition via directly gnawing and indirect regulatory influences, such as changing soil exoenzyme, microbial biomass and diversity, thereby magnifing the effect size of PLE; (3) In soils contaminated with HDPE‐MPs, the toxicity of microplastics to soil biota (especially soil animals) may impede the decomposition rate, resulting in a reduced PLE, and the explanatory power of external variables (UV radiation and decomposer integrality) would also be diminished.

## 2 Materials and Methods

### 2.1 Litter Collection, Soil Animal Pretreatment, and Microplastic Preparation

Litter and soil samples were collected from the Baotianman Nature Reserve in Neixiang County, Nanyang, China (111°47′ ~ 112°04′ E, 33°20′ ~ 33°36′ N), which is situated in the transition zone between the warm temperate and subtropical zones. The sampling site was a subtropical forest dominated by *Quercus variabilis* , a deciduous oak, and accompanied by *L. glauca* (the focal plant of interest). The type of soil was yellow‐cinnamon soil. Prior to the mesocosm experiment, the soil was air‐dried and sieved (2 mm) to eliminate visible debris and stones. The initial pH, organic carbon, total nitrogen, and total phosphorus of the soil were 6.49 ± 0.17, 27.15 ± 0.86, 4.79 ± 0.25, and 1.08 ± 0.18 g/kg, respectively [values are means ± SDs (*n* = 4), see Data S1 file for detailed test process]. Marcescent litters were collected from *L. glauca* branches on December 6, 2019, air‐dried, and sealed in a dark box, and the initial lignin, organic carbon, and total nitrogen contents were determined (Table S6). Forest soils were collected in April, 2020. We chose to introduce the widely distributed earthworms ( *Eisenia fetida* ) and springtails ( *Folsomia candida* ) in treatments that integrate fauna decomposers, as they play crucial ecological roles as saprophytic detritivores (Fountain and Hopkin 2005; Zhu et al. 2018). The earthworms were obtained from Hunan Yiyang Dazu Biological Company, China. A part of the earthworms was firstly preincubated in soils containing HDPE‐MPs for 30 days to stabilize their survivability in such contamination conditions. The remaining individuals underwent a 14‐day acclimatization to familiarize themselves with the laboratory culture environment. Following a 24‐h depuration on moist culture dishes, 32 adults of similar size (16 from polluted environments and 16 from unpolluted environments) were rinsed with distilled water, dried on paper towels, and individually weighed. Two individuals were assigned to each animal addition mesocosm. *F. candida* was collected from the forest soil and cultivated in the laboratory. HDPE‐MPs (Suzhou Win Before New Material Co. Ltd., Suzhou, China) has been established to be toxic to certain soil animals such as earthworms (Boots, Russell, and Green 2019). In our mesocosm experiment, we selected 100 mesh (100–150 μm) HDPE to investigate the impact of microplastics on the decomposition system.

### 2.2 Photodegradation of Marcescent Litter

During the natural photodegradation process, it is UV light that mostly accounts for photodegradation (Austin and Ballaré 2010; Wang et al. 2021, 2023). Accordingly, we selected the 360 nm UV light to construct the artificial weathering chamber to simulate abiotic photodegradation. Exposing to UV radiation has a potential impact on phyllosphere microbes residing in leaf litters, which could become or affect soil microbial decomposers (Fanin et al. 2021). This may lead to disparate decomposition conditions between the photodegraded litters and those kept in the dark. To eliminate this potential impact and solely focus on the abiotic aging, the air‐dried *L. glauca* litters were sterilized by *γ*‐radiation at a 25 kGy dose (Isotope Research Institute of Henan Academy of Sciences). This was done to maintain initial litters in a completely sterile state (both internal and external) without altering their physicochemical properties.

In order to investigate the isolated contribution of radiation on litter decomposition, we divided the sterilized litter into two groups: one kept in the dark and the other was subjected to exposure in the weathering chamber to replicate the abiotic aging process of *L. glauca* during the non‐growing season (ASTM G154 2006 standard). Specifically, the pre‐photodegradation process settled a blackboard temperature of 60°C, which can accelerate the aging process and maintain a relatively sterile environment for the litters. The irradiance was set at 0.68 W/m^2^ for a duration of 72 h, which approximately simulates the natural aging process of nearly 3 months in the local environment. This allowed for the construction of potential litter quality differences that were caused by the abiotic photodegradation.

### 2.3 Biodegradation in Mesocosms

**FIGURE 1:**
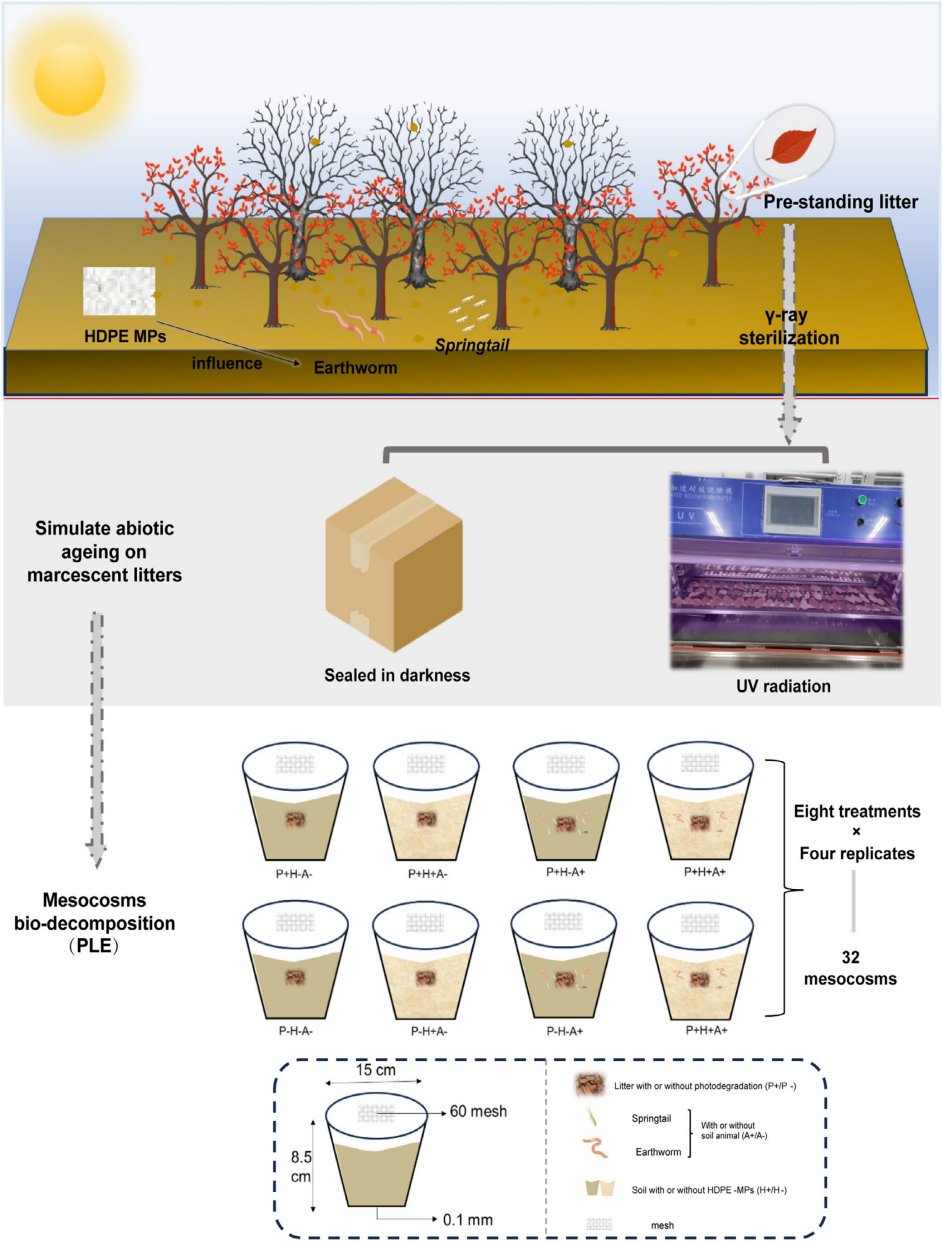
Workflow of constructing mesocosm incubation. Three‐way full factorial design generating eight experimental treatments: With Photodegradation (P+H−A−); No‐photodegradation (P−H−A−); Photodegradation and HDPE‐MPs (P+H+A−); No‐photodegradation and HDPE‐MPs (P−H+A−); Photodegradation and Soil animals (P+H−A+); No‐photodegradation and Soil animals (P−H−A+); Photodegradation and HDPE‐MPs and Soil animals (P+H+A+); No‐photodegradation and HDPE‐MPs and Soil animals (P−H+A+).

The effects of pre‐photodegradation, soil animal addition, and HDPE contamination on the biodegradation process of litter were studied by constructing a full factorial design (Figure 1). The incubation mesocosm unit was constructed using a polypropylene pot (upper diameter 15 cm, bottom diameter 10 cm, height 8.5 cm), with the top covered by a polyethylene breathable mesh (0.42 mm, 60 mesh) to prevent soil animals from escaping, and 0.1 mm holes were settled at the bottom for drainage (Figure 1). Each mesocosm received 400 g of the sieved soil. To test the potential impact of MPs in the active topsoil, half of the mesocosms were uniformly mixed with microplastics at a concentration of 1 g kg^−1^ dry soil (0.1% w/w) (Cheng et al. 2021). The water content was maintained at 60% water holding capacity (WHC) for all the mesocosms. After 7 days of preincubation, half of the polluted and unpolluted mesocosms were supplemented with *E. fetida* (2 individuals per pot) and *F. candida* (150 individuals per pot) and preincubated for an additional 7 days. Similarly, 1 g of the litters subjected to darkness or photodegradation were incubated in each combination of the microplastic × animal treatments by burying them in the surface 5 cm of soil. Eight experimental treatments were set up: P+H−A− (with photodegradation), P−H−A− (non‐photodegradation), P+H+A− (photodegradation and HDPE), P−H+A− (non‐photodegradation with HDPE), P+H−A (photodegradation and soil animals), P−H−A+ (non‐photodegradation with soil animals), P+H+A+ (photodegradation with HDPE and soil animals), P−H+A+ (non‐photodegradation with HDPE and soil animals). Each treatment had four replicates, resulting in a total of 32 mesocosms (Figure 1). These mesocosms were maintained under consistent hydrothermal conditions during the litter incubation (the indoor temperature was 26°C and 60% WHC in every mesocosm).

### 2.4 Determination of Litter Decomposition and Biochemical Characters

After a 90‐day decomposition, we carefully harvested the litters, and the soils closely beneath them were sampled. Soil microbial biomass carbon and nitrogen (MBC and MBN) were determined using the chloroform fumigation‐incubation method (Brookes et al. 1985; Vance, Brookes, and Jenkinson 1987). The litter was carefully cleaned, and a subsample was preserved at −80°C for subsequent analysis of microbial community, while the remaining portion was weighed and dried to a constant weight to determine the mass loss. A subsample of 0.2 g of dried litter was ground into powder using a tissue grinder (Shanghai Jingxin Industrial Development Co. Ltd., China, Tiss‐Basic 24) for chemical determination (lignin, total carbon, total nitrogen). The soil sample was stored at 4°C for enzymatic assay within 5 days. *F. candida* densities were assessed using the Tullgren funnel method (González et al. 2021). Individual *E. fetida* were recovered from each mesocosm, rinsed with distilled water, and their guts were depurated on moist paper towels for 24 h before recording their weight gravimetrically.

Fungi are key decomposers, especially for their ability on lignin decomposition. We assayed the fungal community in the decomposed litters using the high‐throughput amplicon sequencing method (Liu et al. 2021). Total genomic DNA was extracted from litter samples using the CTAB method (Martin and Rygiewicz 2005). The fungal communities were profiled by targeting the ITS1 genes of the ITS1‐1F region, amplified by PCR using the specific primer pairs ITS1‐1F‐F (5′‐CTTGGTCATTTAGAGGAAGTAA‐3′) and ITS1‐1F‐R (5′‐GCTGCGTTCTTCATCGATGC‐3′). PCR products were mixed in equidensity ratios, and the mixture PCR products were purified with universal DNA (Tian Gen, China). Sequencing was conducted on an Illumina HiSeq. 2500 platform (Illumina Inc.). We assembled quality‐filtered reads into amplicon sequence variants (ASVs) using DATA2 (Callahan et al. 2016); the QIIME2 feature‐classifier plugin was then used to align ASV sequences to generate the taxonomy table.

The litter lignin content was quantified using the Klason method (Dence 1992). Litter C and N concentrations were determined using the potassium dichromate external heating method and indophenol blue colorimetry (Nelson and Sommers 2015; Novamsky et al. 1974). The activities of eight soil extracellular enzymes involved in the cycling of litter C and nutrients were determined chromometrically. These enzymes included two oxidative enzymes, phenol oxidase (Pheno) and phenol peroxidase (Perox), and six hydrolytic enzymes that catalyze the cycling of low molecular weight substrates, i.e., cellobiohydrolase (CBH1), β‐1,4‐glucosidase (BG), and β‐1,4‐xylosidase (BX) for C cycling, nitrate reductase (NR) and urease (URE) for nitrogen cycling, and acid phosphatase (ACP) for phosphorus cycling (Vepsäläinen et al. 2001). See Data S1 for detailed assay procedures.

### 2.5 Construction of a Priori Path Model for Litter Decomposition

Correlation analysis was employed to examine the linear relationships between litter mass loss, lignin loss, and enzyme activities associated with soil carbon and nutrient cycling (Figure S6). To enhance comprehension of the intricate relationships of specific key processes that determine litter decomposition, we constructed an a priori model based on the influential factors governing decomposition and conducted quantitative evaluations using structural equation modeling (SEM) (Figure S1). The a priori model encompasses two exogenous binary variables (abiotic photodegradation and soil animals), along with two endogenous explanatory modules, i.e., the recalcitrant lignin loss and soil enzymatic activity, to comprehensively assess their respective impacts on litter mass loss. Based on prior research findings, we supposed that the extent of litter mass loss may be influenced by the direct exposure of leaf litter to irradiation, as well as its subsequent impact on lignin decomposition. Additionally, soil‐dwelling organisms can directly influence litter decomposition through processes such as transportation and consumption while also indirectly affecting microbial activities that govern enzyme efficacy and lignin degradation (Dick 2015). For the characterization of soil microbial decomposition activities, we comprehensively assessed the soil enzyme activities beneath the litter. To improve the a priori model, we employed Principal Component Analysis (PCA) to reduce the dimensionality of enzymes and capture their comprehensive interplay while considering their actual influences on the other endogenous variables. Overall, the first two components accounted for 29.1% and 20.66% of the enzymatic variance (Figure S2). The first component (PC1) was mostly associated with CBH1 (*ρ* = −0.636, *p* < 0.001); Component 2 (PC2) was positively correlated with CBH1 (*ρ* = 0.454, *p* = 0.009) and BG (*ρ* = 0.594, *p* < 0.001) (Table S2). Notably, BG and CBH1 were orthogonal with each other and demonstrated a strong fit with the other endogenous variables when constructing SEM (GIF > 0.9, *p* > 0.05, RMSEA < 0.05). Therefore, we used CBH1 and BG to construct the enzymatic activity module in the SEM (Figure S1 and Table S7). The a priori model was applied independently to both the HDPE‐MPs polluted and unpolluted systems. A comparative analysis of the pivotal pathways between these two systems was conducted to elucidate the impact of microplastic on the litter decomposition process.

### 2.6 Data Calculation and Statistical Analysis

Litter mass, lignin, total C and N losses were calculated as follows:

(1) $$M\;\left(\%\right)=\left(M_{t}-M_{0}\right)/M_{0}\times 100$$

(2) $${\mathrm{CNL}}_{\text{loss}}\left(\%\right)=\left[\left(M_{0}\times {\mathrm{CNL}}_{0}-M_{t}\times {\mathrm{CNL}}_{t}\right)/\left(M_{0}\times {\mathrm{CNL}}_{t}\right)\right]\times 100$$

where *M* represents mass loss, *M*_*0*_ and *M*_*t*_ denote the litter mass at initial and after 90 days of decomposition, respectively; *CNL*_*loss*_ represents the loss of lignin, total C, and N, while *CNL*_*0*_ and *CNL*_*t*_ signify their initial and end concentrations.

Recent studies had pinpointed the effects of microplastics on soil animals and microbes (Huerta Lwanga et al. 2016; Anbumani and Kakkar 2018; Huang et al. 2019). In order to investigate the potential impact of microplastic on PLE through their influences on decomposers, we compared the PLEs in two contrasting systems (with or without microplastic pollution) by examining the differences between soil decomposers (animals + microbes vs. microbes only). For the soil animal addition treatments, animal activity was assessed by population density and the weight growth rate (W%) of their biomass:

(3) $$W\;\left(\%\right)=\left(W_{t}-W_{0}\right)/W_{0}\times 100$$

where *W* represents animal weight growth, *W*_*0*_ denotes the initial weight of animal biomass, and *W*_*t*_ signifies the final weight after incubation.

Specifically, we quantified the impact of soil animals on the PLE in HDPE‐MPs polluted and unpolluted systems. The PLE on subsequent bio‐decomposition in soil was calculated by determining the response ratio of mass loss or element release between litter that experienced UV exposure and litter kept in darkness (Formula 4). The impact of soil animals on PLE (*A*_*PLE*_) was quantified by the difference between *PLE*_*M.A*_ and *PLE*_*M*_ (Formula 5).

(4) $$\mathrm{PLE}=\ln\left({\text{loss}}_{P}/{\text{loss}}_{\mathrm{NP}}\right)$$

(5) $$A_{\mathrm{PLE}}={\mathrm{PLE}}_{M.A}-{\mathrm{PLE}}_{M}$$

where *loss*_*P*_ and *loss*_*NP*_ represent the mean values of litter mass loss and element release for photodegradation (P) and no‐photodegradation (NP), respectively; *PLE*_*M.A*_ and *PLE*_*M*_ refer to the bio‐decomposition with and without soil animals, and the magnitude of their difference is denoted by *A*_*PLE*_, which was used to assess the soil animal's regulatory influences on PLE.

Fungal diversity in the decomposed litters was evaluated by calculating the Shannon entropy (*H*):

(6) $$H=-\sum_{i=1}^{S}p_{i}\log_{2}\left(p_{i}\right)$$

where *S* is the richness of detected ASVs, and *p*_*i*_ signifies the relative abundance of ASV_i_ in the fungal community.

All analyses were conducted using R version 4.3.2 (R Development Core Team, https://www.r‐project.org). Three‐way ANOVA was employed to examine the effects of photodegradation, soil animals, HDPE‐MPs, and their interactions on litter mass loss, chemical changes (lignin content, C and N loss, lignin loss, C:N ratio, and lignin:N ratio), and soil exoenzyme activities (CBH1, cellobiohydrolase; BG, β‐1,4‐glucosidase; BX, β‐1,4‐xylosidase; NR, nitrate reductase; URE, urease; ACP, acid phosphatase; Pheno, phenol oxidase; Perox, phenol oxidase). A series of t‐tests were used to compare the experimental variables between two distinct groups while controlling for other factors. The soil enzyme activities were regarded as microbial kinetic functions on the biodegradation process within contrasting soils, and the experimental impact on them was evaluated using semi‐parametric permutational multivariate ANOVA (PERMANOVA) and PCA. Package “GGally” was used for multivariate variable regressive analysis, package “vegan” was used for PERMANOVA and PCA, and package “lavaan” was used for SEM analyses.

## 3 Results

### 3.1 Effect of UV Irradiation on Litter Quality During the Marcescence Process

**FIGURE 2:**
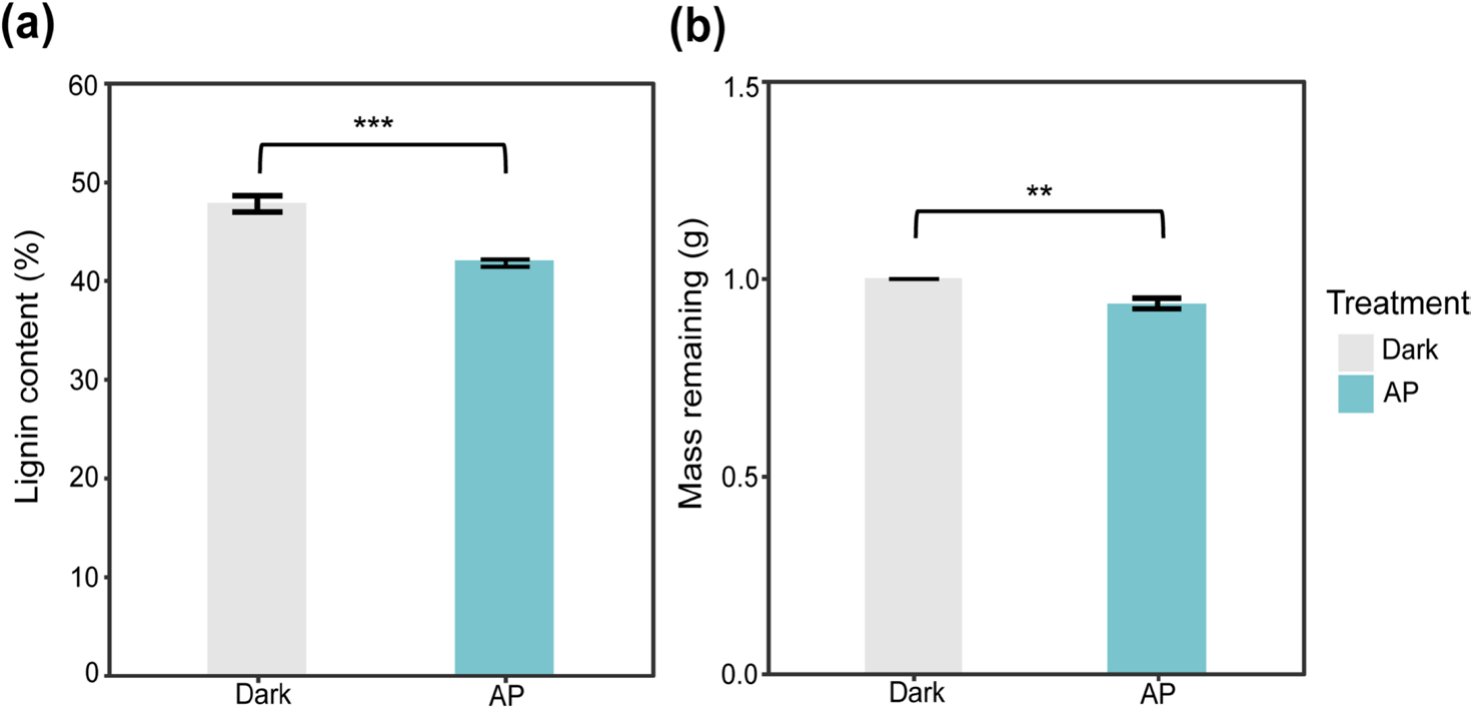
Different influences of abiotic aging (AP, simulates marcescent photodegradation process, treated in the UV accelerated weathering tester box) and darkness (Dark, treated in a sealed dark box) on (a) mass remaining and (b) lignin content of freshly senescent *L. glauca* litters. Values are means ± SD (*n* = 4). Asterisks indicate significant differences between groups (**0.001 < *p* < 0.01, ***p < 0.001).

The lignin content of marcescent *L. glauca* litter was significantly decreased by 12.57% after the abiotic aging process (*t* = −16.15, *p* < 0.01, Figure 2a and Table S3). Additionally, photodegradation also led to a litter mass loss of 6.13% compared to the dark ones (*t* = −11.88, *p* < 0.05, Figure 2b and Table S3). Meanwhile, there was a significant increase in litter total N content of 22.46% (*t* = 6.94, *p* < 0.001, Figure S3 and Table S3), but no significant difference in litter total C content (*t* = −0.88, *p* > 0.05, Figure S3 and Table S3). The process of simulated marcescent litter aging significantly decreased litter C:N and lignin:N by 19.49% (*t* = −6.25, *p* < 0.001) and 28.61% (*t* = −9.76, *p* < 0.001, Figure S3 and Table S3), respectively. Therefore, the litter quality has been significantly changed by abiotic photodegradation.

### 3.2 Litter Decomposition and Chemical Changes

**FIGURE 3:**
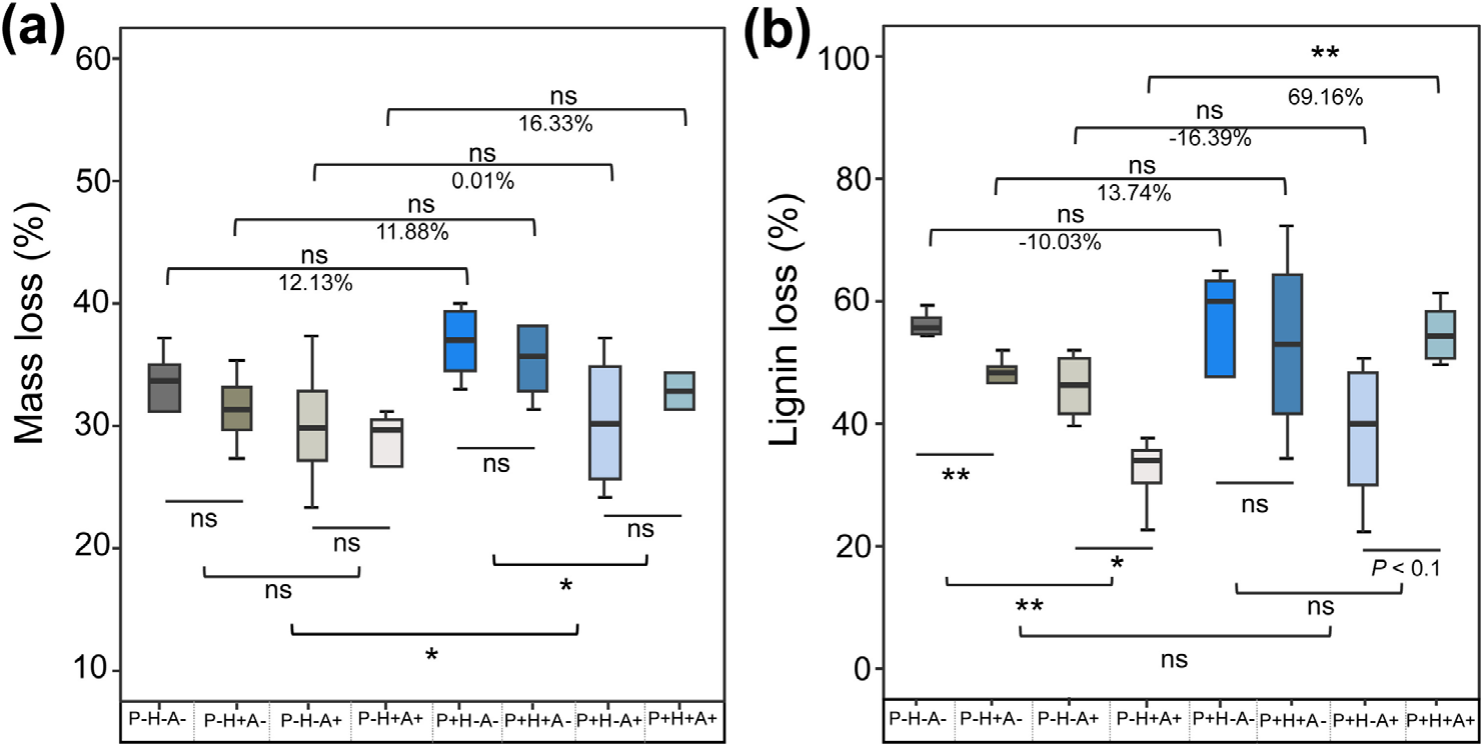
Interactive influences of photodegradation, soil animal, and HDPE‐MPs on (a) litter mass loss and (b) litter lignin loss after 90 days of incubation. Different colors represent various treatments involving P+H−A−, with photodegradation; P−H−A−, non‐photodegradation; P+H+A−, photodegradation and HDPE; P−H+A−, non‐photodegradation and HDPE; P+H−A, photodegradation and soil animals; P−H−A+, non‐photodegradation and soil animals; P+H+A+, photodegradation and HDPE and soil animals; P−H+A+, non‐photodegradation and HDPE and soil animals. Asterisks indicate significant differences between groups (*p < 0.05, **p < 0.01).

After 90 days of biotic decomposition, all mesocosms had a similar soil pH of 5.18 ± 0.02 (mean ± SE). Overall, in all incubations, *L. glauca* litters experienced a mass loss of 31.88%, with a lignin loss of 47.17%, a decrease in lignin content of 22.83%, a net C loss of 37.47%, and a net increase in nitrogen (N) immobilization of 17.98% (compared with the status at initial incubation, Figure 3, Figure S4, and Table S4). The three‐way ANOVA showed that the main effect of pre‐photodegradation significantly accelerated litter mass and C losses. Controlling for other treatments, photodegraded (post‐abiotic aging) litter generally tended to experience faster decomposition than the dark‐treated litters, although these tendencies were mainly statistically insignificant (Figure 3, Figure S4, Table 1, and Table S4). Concretely, pre‐photodegradation increased litter mass and C loss by 12.13% and 9.03% (P+H−A− vs. P−H−A−), 0.01% and −1.67% (P+H−A+ vs. P−H−A+), 11.88% and 15.30% (P+H+A− vs. P−H+A−) and 16.33% and 18.02% (P+H+A+ vs. P−H+A+), respectively. Additionally, litter lignin loss was also increased by 13.74% (P+H+A− vs. P−H+A−) and 69.16% (P+H+A+ vs. P−H+A+).

**TABLE 1:** Statistics of the three‐way ANOVA used to analyze the effects of litter post‐photodegradation, HDPE microplastic, and soil animals (with or without) on the litter mass, lignin loss, C and N losses, as well as litter C:N and lignin:N ratios. Significant (*p* < 0.05, bold) or marginally significant relationships (*p* < 0.1, bold italic) are highlighted.

Factors	df	Mass loss		Lignin loss		C loss		N loss		C: N		Lignin: N	
		*F*	*p*	*F*	*p*	*F*	*p*	*F*	*p*	*F*	*p*	*F*	*p*
P (photodegradation)	1	4.83	**0.04**	0.91	0.35	3.42	***0.08***	0.68	0.42	0.01	0.94	1.70	0.21
H (HDPE‐MPs)	1	0.18	0.68	0.31	0.58	0.34	0.56	4.08	***0.06***	0.78	0.39	0.17	0.69
A (soil animals)	1	5.53	**0.03**	5.47	**0.03**	6.50	**0.02**	0.81	0.38	2.34	0.14	5.97	**0.02**
P × H	1	0.66	0.43	1.14	0.30	0.33	0.57	2.43	0.13	1.60	0.22	0.17	0.68
P × A	1	0.18	0.68	0.72	0.40	0.25	0.62	2.89	0.10	1.78	0.20	3.65	***0.07***
H × A	1	0.25	0.62	0.04	0.84	0.15	0.70	6.86	**0.02**	4.32	**0.05**	2.92	0.10
P × H × A	1	0.43	0.52	6.52	**0.02**	1.19	0.29	0.32	0.58	0.33	0.57	3.12	***0.09***

Soil animals mainly had an inhibitory impact on litter decomposition. Animal addition decreased litter mass, lignin, and C losses, as well as decreased litter lignin: N ratio. Specifically, litter mass loss was decreased by 23.33% (P+H−A+ vs. P+H−A−) and 6.67% (P−H−A+ vs. P−H−A−), and lignin loss was decreased by 23.68% (P+H−A+ vs. P+H−A−) and 24.07% (P−H−A+ vs. P−H−A−; Figure 3 and Table 1). When interacting with other factors, animal addition had additional controls on litter chemical changes regarding litter quality and nutrient release, such as N loss, C:N and lignin:N ratios (Figure S4).

High‐density polyethylene microplastics only exerted a marginal significance on litter N loss. However, when interacting with the other two factors, it exerted an interactive impact on lignin loss (*F* = 6.52, *p* < 0.05, Table 1). There was a consistent depression of lignin degradation in the non‐photodegraded litters, regardless of the presence of animals, and the inhibitory effect of animal addition was magnified when soil was polluted. However, for the photodegraded litters, HDPE‐MPs didn't show any depressive effect and could even increase lignin loss within the animal addition treatments (Table 1 and Figure 3).

### 3.3 Characteristics of Decomposers

**FIGURE 4:**
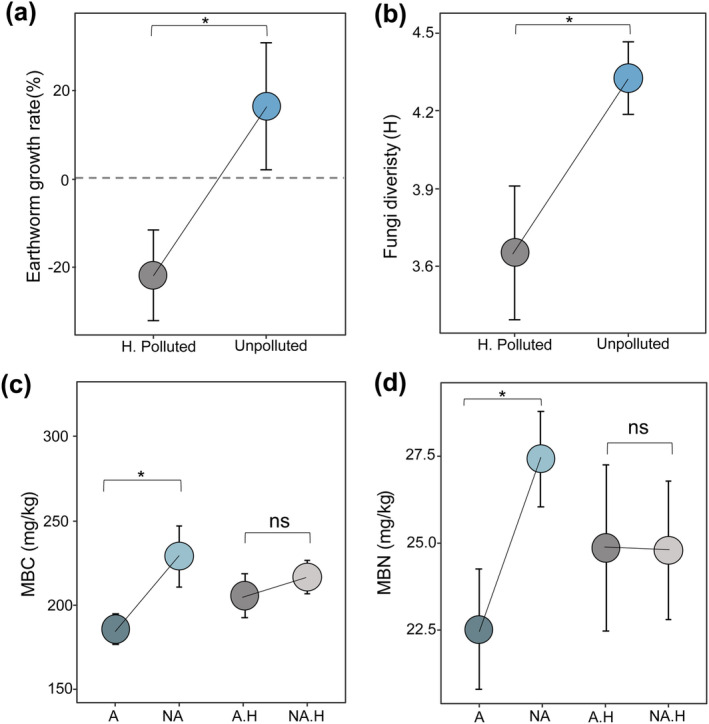
Decomposer performances and interactions after 90 days of bio‐incubation. (a, b) Show the relative changes in (a) earthworm weight growth rate (%) and (b) litter fungi diversity (H) between mesocosms with HDPE‐MPs absent (unpolluted) or present (H. polluted); (c, d) Show comparisons of soil animals' impact on (c) soil microbial biomass carbon (MBC) and (d) soil microbial biomass nitrogen (MBN) under HDPE polluted environments or not, A and NA: with and without soil animals in unpolluted mesocosms, A.H. and N.H. With and without soil animals in polluted soils. Values and error bars represent means ± SD, asterisks indicate significant differences between contrasting treatments (*p* < 0.05), and ns denotes non‐significant (*p* ≥ 0.05).

There were no significant differences in *F. candida* biomass or density between the experimental treatments, but the weight growth rate of the *E. fetida* significantly differed between the unpolluted and HDPE‐MPs polluted systems. Specifically, compared to the initial weight, the weight of earthworms increased by 16.68% in unpolluted systems (*p* < 0.05, Figure 4a, Unpolluted), whereas it decreased by 21.62% in the polluted soils (*p* < 0.05, Figure 4a, H. Polluted). Meanwhile, HDPE‐MPs also significantly decreased the fungi diversity in decomposed litters (*p* < 0.05, Figure 4b). Furthermore, for the unpolluted mesocosms, soil microbial biomass C and N (MBC and MBN) were significantly lower in the animal addition soils than in the non‐animal ones (A vs. NA, Figure 4c,d), but in HDPE‐MPs polluted soils, there was no significant difference between the animal treatments (A.H vs. NA.H, Figure 4c,d). PERMANOVA showed that soil animals, HDPE‐MPs, and their interactive effect had significant consequences with soil enzymatic variations (Figure S2 and Tables S1 and S5).

### 3.4 Context Dependency of PLE

**FIGURE 5:**
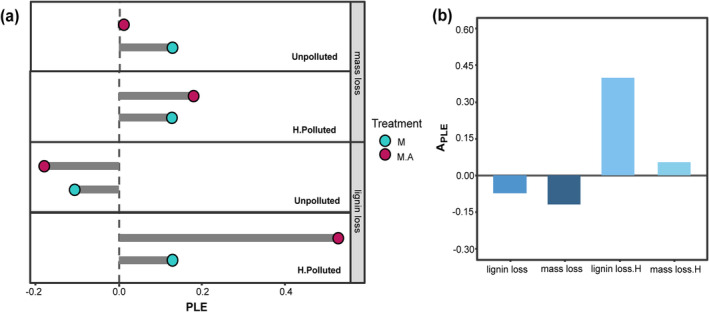
Effect size of the legacy effect of pre‐photodegradation (PLE) on subsequent bio‐decomposition. (a) In two contrasting environments that contained HDPE‐MPs (H. polluted) and not (unpolluted), and with (M.A.) or without (M) the impact of soil animals, the PLEs on mass loss (*PLE*_*massloss*_) and lignin loss (*PLE*_*ligninloss*_) were shown. (b) Soil animal's regulatory influences on PLE (*A*_*PLE*_) of lignin loss and mass loss, H denoted incubated in soils contaminated by HDPE‐MPs.

When soil animals were not incorporated in the decomposition mesocosm, there was mainly a positive PLE observed on litter mass (0.13), lignin (0.01), and C (0.14) losses (Figure 5a and Figure S5a). However, the addition of soil animals had contrasting effects on PLEs under different environments. In unpolluted systems, animal addition decreased the PLE. Specifically, *PLE*_*massloss*_ decreased from 0.13 to 0.01, *PLE*_*Closs*_ decreased from 0.09 to −0.02, and a negative *PLE*_*ligninloss*_ of −0.11 was also magnified to −0.18 (Figure 5a and Figure S5a, Unpolluted). However, in soils polluted with HDPE‐MPs, animal addition resulted in an increase of PLE, made *PLE*_*massloss*_ increased from 0.13 to 0.18, *PLE*_*Closs*_ increased from 0.14 to 0.17, and *PLE*_*ligninloss*_ increased from 0.13 to 0.53 (Figure 5a, H. polluted). Hence, the soil animal's regulatory influences on PLE exhibited a negative *A*_*PLE*_ in unpolluted mesocosms but a distinct positive *A*_*PLE*_ in polluted soils (Figure 5b and Figure S5). As a result, HDPE‐MPs significantly increased the PLEs when soil contained animals.

### 3.5 Decomposition Path Ways

**FIGURE 6:**
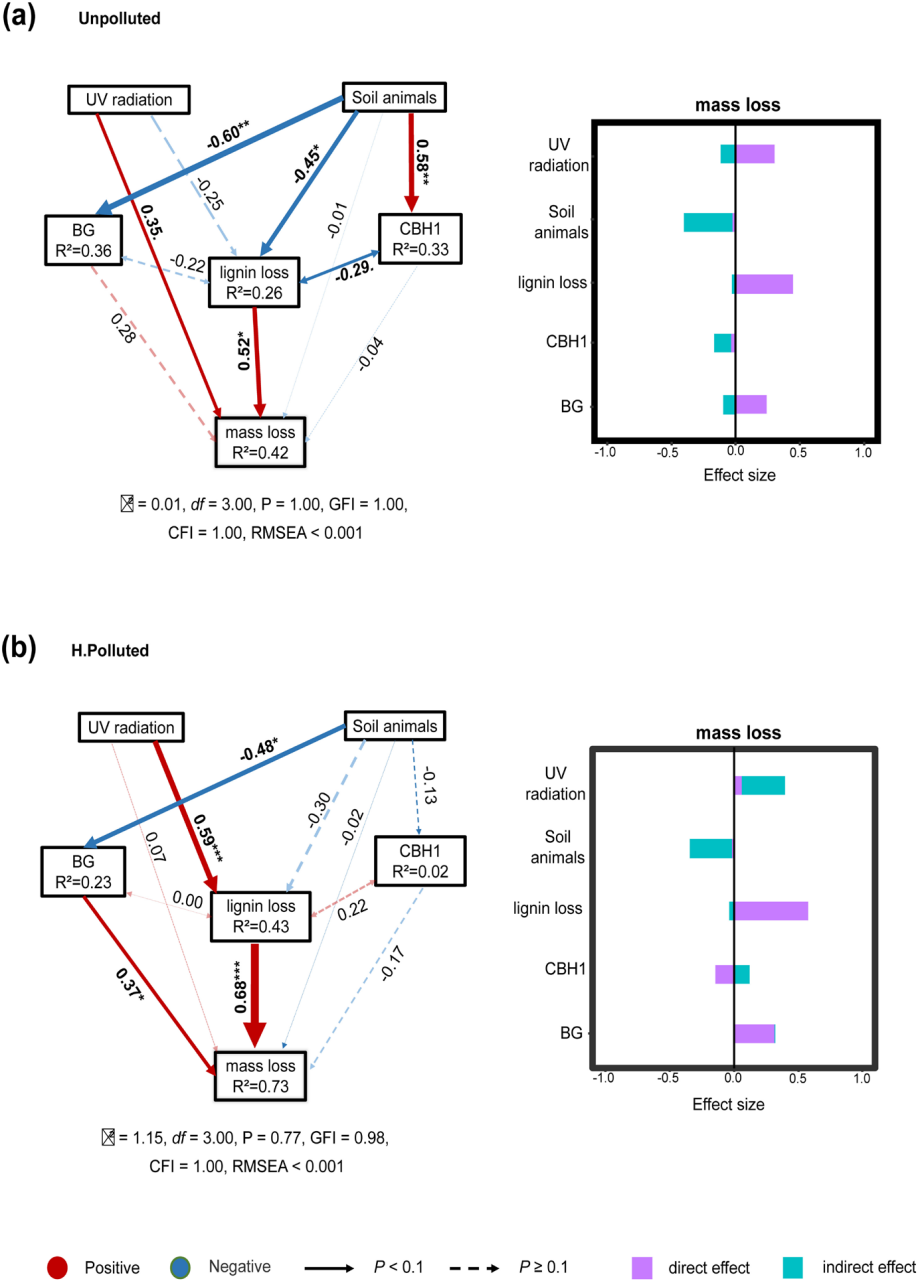
Structural Equation Models (SEMs) on the effect of UV radiation and soil animals on litter mass loss in (a) unpolluted and (b) HDPE‐MPs polluted (H. Polluted) mesocosms via influential paths on litter lignin loss and soil enzyme activities (BG: β‐glucosidase, CBH1: Cellobiohydrolase). Path coefficients and effect sizes are standardized. Direct and indirect effects were calculated for each explanatory variable on litter mass loss (shown on the right). Solid red and blue arrows represent significant (*p < 0.05, **p < 0.01, ***p < 0.001) and marginally significant relationships (‘.’, *p* < 0.1), and dashed arrows represent non‐significant paths (*p* ≥ 0.1). The width of arrows denotes the path coefficients, and *R* ^2^ denotes the explanatory proportion of variances explained for each endogenous variable. The statistical data of goodness of fit for each SEM are given (χ^2^, *df*, *p*‐value, GFI, CFI, RMSEA).

Structural equation model showed that the *a prior* model effectively accounted for the litter decomposition. All explanatory variables had similar net contributions on mass loss, yet the explanatory power was relatively lower in the unpolluted mesocosms (*R* ^2^ = 0.42) than in the HDPE‐MPs polluted soil (*R* ^2^ = 0.73), indicating more complicated processes in the former that remained unexplained (Figure 6).

The significant impact of lignin loss on mass loss was particularly pronounced in the HDPE‐MPs polluted environment. The abiotic aging (UV radiation) of the process had a facilitating net effect on subsequent mass loss in both contaminated and uncontaminated conditions, but their action paths differed. In the unpolluted environment, UV radiation only had a minimal direct influence on litter mass loss (Figure 6a, Unpolluted). Yet in HDPE‐polluted soils, the positive impacts of UV radiation on litter mass loss were indirectly modulated through its impact on the degradation of litter lignin (Figure 6b, H. Polluted).

The addition of soil animals only indirectly exerted a negative influence on litter mass loss; the pathway varied depending on the contamination environment. In an unpolluted environment, soil animals influenced all endogenous explanatory variables, and their indirect depressive effect on litter mass loss primarily occurred by directly reducing litter lignin loss or indirectly by accelerating cellobiohydrolase (CBH1) activity, which exhibited a negative correlation with lignin loss (Figure 6a). However, in the HDPE‐polluted system, the indirect depressive effect size of soil animals on litter mass loss was smaller and primarily occurred through the reduction of β‐glucosidase (BG), which was positively correlated with mass loss, with no influences on lignin loss (Figure 6b).

## 4 Discussion

### 4.1 Legacy Effect of Abiotic Photodegradation Depends on Early Phase Litter Lignin Changes

Marcescence is very common among plants; e.g., a recent study of European flora shows that most of the plants have some proportion of marcescent litter (Mudrák et al. 2023). Thus, the photofacilitation in ecosystems might be widely overlooked. In deciduous and semi‐deciduous forests, a substantial amount of litter accumulates during leaf falling and non‐growing seasons. *L. glauca* exhibits marcescence throughout the non‐growing season, with incomplete development of cellular lysis in the abscission layer zone of its withered leaves (Figure S9a–c). This results in a unique scene where marcescent leaves hang on before eventually shedding and falling into soil in spring (Figure S9d,e). When the forest contains marcescent shrubs, dungarungas, or macrophanerophytes like *L. glauca* , they can replenish the soil decomposition system with fresh and photodegraded leaf litter during forest germination in spring. This reminds us that such temporal differentiation can prolong leaf return and improve litter nutrient use efficiency: soil decomposition of these litters starts at the beginning of the growing season, but not in the no‐litter‐shortage falling season. This phenomenon could regulate the entire soil CO_2_ flux and might even generate an overlooked carbon sequestration mechanism through the prolonged decomposition process, reduced nutrient leaching, higher carbon use efficiency, and so on.

Lignin is believed to be a crucially recalcitrant substrate that greatly restrains the microbial enzymatic decomposition (Swift, Heal, and Anderson 1979). It is typically entangled with cellulose and hemicellulose in the cell wall. Photochemical aging has the ability to directly break down organic matter through abiotic mineralization of complex lignin, which can absorb short‐wave radiations (UV and blue visible light) (Austin and Ballaré 2010). This process can indirectly benefit microbes by producing labile organic matter from the recalcitrant compounds (Berenstecher et al. 2020). In our study, the photodegradation process led to a significant improvement of litter quality within the artificial aging chamber (Figure 2 and Figure S3), which is in accordance with our first hypothesis. Recent studies have also confirmed the accelerating impact of UV photodegradation on biotic decomposition, i.e., the positive PLE, in forests when solar radiation is sufficient (Wang et al. 2020; Jiang et al. 2023). The observed decrease in lignin content (12.57%, Figure 3) was within the ranges of most field photodegradation studies (Austin, Méndez, and Ballaré 2016; Jiang et al. 2022). Comparing with these studies, the magnitude of photo‐acceleration of litter mass loss during soil decomposition is slightly less in our experiment (11.81% overall and 7.63% in unpolluted treatments; Figure 3a). This may be due to the restricted control of aboveground abiotic photodegradation in our experiment; all litters were *γ*‐radiation sterilized, subjected to aging temperature at 60°C, and kept dry), while in nature the non‐sterile photodegradation also includes biochemical processes that will accelerate further litter decomposition (García‐Palacios et al. 2016; Fanin et al. 2021). Besides, we did not incorporate moisture fluctuation in the chamber to keep the abiotic state equally compared to the sealed dark ones, while irradiation in nature also promotes the leaching loss in humid conditions (Lin et al. 2018; Marinho et al. 2020). Nevertheless, the recent utilization of artificial weathering chambers offers an efficient means to assess the environmental aging influences such as the photodegradation. This technique presents an opportunity to determine the pure abiotic photochemical aging impact on plant litter and its mechanistic cascading effect on further bio‐decomposition.

Litter quality controls litter decomposition hierarchically at local to global scales and affects not only the rate of litter mass loss but also the patterns of nutrient release (Cornwell et al. 2008; Zhang et al. 2008; Makkonen et al. 2012; Mooshammer et al. 2012). Initial litter quality alone can determine the trajectory of decomposition (Manzoni et al. 2010). Fresh litter contains much free and easily decomposable organics, and generally, its decomposition experiences nutrient leaching, immobilization, and net release (Hall et al. 2020; Klotzbücher et al. 2011). In the early phase of decomposition, available organic C, N, and other nutrients were rapidly consumed by microorganisms. As litter quality deteriorates, K‐strategy fungi grow their hyphae to degrade large benzene chains and release the lignin‐wrapped cellulose and other smaller carbon components, and N is enriched on litter residues in the form of mycelium, preparing for further net mineralization (Zhou et al. 2021). Thus, according to traditional theories, the recalcitrant lignin mainly remains to be decomposed in the later phase by fungi, e.g., visible as white areas colonized by white rot fungus (Keyser, Kirk, and Zeikus 1978; Osono and Takeda 1999). Accordingly, lignin loss would compose a small part of the total fresh litter mass loss. However, in our experiment, the lignin loss reached 45.23% and 49.12% after soil bio‐decomposition for the dark and irradiation‐treated fresh litters, respectively (Figure 3b). This indicates that lignin degradation was a very important component during the earth phase litter decomposition. It has been demonstrated that lignin decomposition is controlled by the availability of easily decomposable carbon (Hall et al. 2020). Compared to old decomposed residuals, the lignin in fresh litters would decompose faster in the initial phase through the co‐metabolism in fresh litters (Klotzbücher et al. 2011; Hall et al. 2020). In line with this conceptual framework, our mesocosm experiment exclusively incubated the newly senescent litters, which possessed ample, easily decomposable C, leading to a rapid decomposition of lignin. This rapid decomposition might further promote the exposure of other degradable components to microbial decomposers and generate positive feedback in the early phase. These elucidate the positively correlation observed between litter mass loss and lignin degradation in our experiment. Meanwhile, the lignin content decreased to 37.33% (from initial 47.75%) and 31.41% (from initial 41.25%) for the dark and irradiation‐treated litters and significantly differed from each other (*F* = 5.453, *p* = 0.026; Figure 3), which suggests that the abiotic aging of marcescent litters can enhance their subsequent decomposition process by altering the trajectory of lignin content. These findings are also consistent with our first hypothesis. Therefore, when modeling forest biogeochemical cycles to accurately assess C and nutrient dynamics, it is important to incorporate the relative significance of photochemical mineralization and its long‐term impact on litter decomposition, as well as how they might respond to environmental changes.

### 4.2 Soil Animal and HDPE‐MPs Pollution Interactively Regulate Actual PLE

Besides microbial decomposers, soil animals are highly relevant to subterranean ecological processes, and they generally display a positive effect on litter decomposition globally (García‐Palacios et al. 2013). Soil animals can not only alter soil physicochemical structure but also possess intense interaction with soil microbial community in regulating the decomposition process (Dick 2015; Lee and Foster 1991; García‐Palacios et al. 2013). In contrast to our second hypothesis, soil animals mainly exerted a negative influence on litter decomposition in our experiment (Figures 3, 5, and 6). In fact, the net effect of soil animals on litter decomposition is a synergistic process that depends on decomposer interactions (Nielsen 2019). For example, the direct gnawing of litter by macro‐ and meso‐detritivores such as earthworms, mites, and springtails can increase the specific surface area of litter exposed to microbial decomposers, which is the main causality of accelerated decomposition, while they can also depress microbial community through feeding on them (Räty and Huhta 2003; Scheu, Ruess, and Bonkowski 2005). These detritivores selectively consume bacteria, fungi, and other microorganisms, thereby regulating microbial activities and affecting their decomposition of organic substrates (Tordoff, Boddy, and Jones 2008). Interestingly, we did not find any obvious sign of mechanical fragmentation caused by soil animal feeding on the litters (Figure S8). This indicated that the fresh litter had little attraction for the employed springtails and earthworms. Thus, it is mainly the microbial‐mediated degradation processes, but not the direct consumption of soil animals, that drive the litter decomposition, as well as any observed changes of PLE (Figure 6). Furthermore, animal addition significantly decreased soil microbial biomass (MBC and MBN) in the unpolluted mesocosms (Figure 4c,d). After 90 days of decomposition, litter mass lost 31.88% while the litter body structure remained relatively intact (Figure 3 and Figure S8), verifying an early phase of decomposition. Hence, we suspected that the active decomposition of fresh litter in our experiment is mainly performed by the microbes, which are preyed upon and depressed by the soil animals. The detritivores during this stage might just treat the fresh litters as their “residence” but not “food”. Besides, *a prior* model analysis found no directive effect of soil animals on litter decomposition; the negative influence was indirectly performed through decreased lignin degradation and changed soil enzymatic activities (Figure 6). This is consistent with the aforementioned co‐metabolism of fast lignin loss of fresh litters, and the predation of detritivores on microbial residents would inhibit the decomposition of macromolecular organic matters. Furthermore, soil animals can also regulate soil physicochemical characteristics, crumb structures, and diffuse litter and microorganisms through their movement in the soil. We found vermicompost spread everywhere in the animal addition treatment (Figure S7), and some litters were found to be transported and buried in the bottom of the mesocosm, which may reduce the lignin exposure to efficient decomposer fungi (Lee and Foster 1991).

**FIGURE 7:**
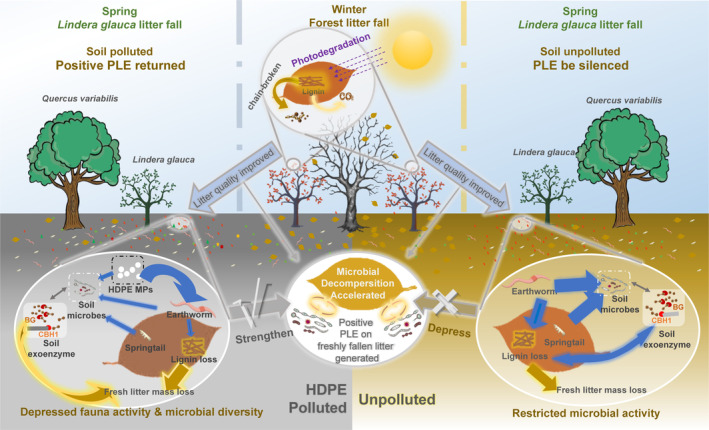
Schematic of marcescence photodegradation and its legacy effect on understory litter decomposition in deciduous forest. Senescent leaves of *L. glauca* hanging on branches throughout the entire autumn and winter before they shed and fall into soil in spring. The legacy effect of photodegradation (PLE) accelerates microbial decomposition of freshly shed litters because preceding aging in branches has softened the recalcitrant lignin during the non‐growing season, making the litters more decomposable. However, decomposer interactions, especially the top‐down regulatory function of soil animals on microbial activity, can retard litter decomposition rate and thus partly depress the PLE to offset potential C release changes. Emerging microplastics can additionally be accumulated in soil via direct atmospheric deposition in the non‐growing season, which will depress soil animal activity and microbial diversity, make microbial decomposers become more sensitive and dependent on the litter quality changes (e.g., increased by photodegradation), and thus strengthen the positive PLE. Arrows denote the strength of relationships in soil; orange arrows indicate positive relations, and blue arrows indicate negative relations.

Consequently, a more complete decomposer structure (i.e., with both microbes and soil animals) can inhibit the positive PLE due to their actual net regulatory functions on the accelerated litter decomposition (hereafter, offset effect). This may be a neglected mechanism that biodiversity influences ecosystem function such as carbon sequestration (Zhang et al. 2013). In line with this new assumption, we found that PLEs on microbial decomposition (C, lignin, and mass losses) were stabilized around a mean of 0.1 in all no‐animal mesocosms (Figure 5 and Figure S5). However, adding *E. fetida* and *F. candida* resulted in a decrease of the positive PLE to nearly disappear, thereby contradicting our second hypothesis. Overall, the PLEs exhibited a distinct inconsistency in decomposer × pollution interactions (Figure 5 and Figure S5). This highlights that incorporating photodegradation in forest biogeochemical cycles is also dependent on soil environment. For instance, soil animals could enhance, but not depress, the PLE in and only in the HDPE‐polluted environment, and HDPE‐MPs greatly enhanced the effect size of PLE for the animal addition treatments (Figure 5 and Figure S5). These findings contradict our third assumption. Therefore, we have developed a conceptual model to illustrate how photodegradation and its legacy effect on marcescent litter decomposition are regulated by soil decomposers in deciduous forests and how emerging microplastics would disrupt the previously established equilibrium of litter‐decomposer interaction (Figure 7).

### 4.3 A Conceptual Model Regarding the Context Dependence of PLE

The acceleration of microbial decomposition of recently fallen marcescent litters in spring results in a positive PLE. The core mechanism of such PLE is that abiotic aging (pre‐photodegradation) increased the litter quality for microbial decomposers, leading to fast lignin and mass losses, at least in the early phase of litter decomposition. However, diverse soil organisms drive ecosystem multifunctionality (Delgado‐Baquerizo et al. 2017, 2020). Soil animals could exert an offset effect on the accelerated litter decomposition as we found, thus the actual PLE is regulated by decomposer interactions and their multifunctionality (Figure 5). Also, the regulatory effects exerted by soil animals on microbial functions exhibit significant variability, contingent upon their actual activities, e.g., grazing intensity or specific top‐down cascading effect (Conti et al. 2020; Crowther et al. 2011; Liu et al. 2019; Wang et al. 2022). Due to the considerable influence of anthropogenic pollutants, such as microplastics, on animal activities, the regulatory effect may become more complex.

Ingestion of MPs has been observed to have detrimental impacts on the soil animal growth, reproduction, and physiological activities, specifically by impairing their endocrine, digestive, and immune systems (Boots, Russell, and Green 2019; Jiang et al. 2020; Li et al. 2021). Accordingly, our study revealed a significant suppression of *E. fetida* by HDPE‐MPs, indicating a potential alteration in their regulatory functions on microbial decomposers. However, no significant influence of HDPE‐MPs on *F. candida* biomass was observed, possibly due to the relatively large particle size of HFPE in this study, making it difficult for *F. candida* to directly ingest (Kim and An 2020). However, it should be noted that MPs at a size of 100–200 μm can also be transferred by *F. candida* , resulting in significant alterations to soil functions (Luo et al. 2023). Therefore, compared to the unpolluted environment, soil animals and all the decomposers might have exerted varying influences on the decomposition system. In polluted mesocosms, the depressed soil animals no longer exert significant influence on soil MBC and MBN and even might help to stimulate a greater positive PLE (Figures 4c,d, 5 and Figure S5). However, even the fungal diversity in decomposed litters was significantly lower in polluted soil than in the unpolluted ones (Figure 4b), we didn't find any significant relationships between the fungi community and litter decomposition; this might be due to the fact that fungi act more as constrained mediators rather than controllers of chemical changes during litter decay (Maillard et al. 2023). All these findings warrant further explorations to elucidate the underlying mechanisms that govern the specificities of specific influences of MPs on substrate‐decomposer and decomposer‐decomposer interactions.

The primary causality of PLE is the assumed indirect effect of irradiation on litter mass loss through accelerating microbial decomposition of the recalcitrant lignin. SEM analysis revealed that soil animals could regulate microbial litter decomposition indirectly through decreasing lignin degradation and changing soil enzymatic activities (Figure 6). The negative effect size on initial litter mass loss was greater in unpolluted environments (Figure 6a). Considering animals had more influences on soil enzyme activities and microbial biomass in unpolluted soils, it also partially explained why the stable PLE disappeared when animals were added in these mesocosms (Figure 5 and Figure S5). The causality process of lignin change would serve as a critical link relevant to the divergent outcomes of PLE in various environments. Accordingly, during early phase decomposition, the pre‐irradiated litters may display different lignin change characteristics under contrasting soil environments. This is also verified by our findings that there were larger changes in lignin content (less final content) in MP‐polluted soils than in the unpolluted ones (*p* = 0.032), and greater lignin loss for the former (53.92%) than the latter (44.31%) (marginally significant, *p* = 0.065; Figure 3b). Besides, the most obvious difference between the two SEMs is that the exogenous explanatory variable UV (i.e., changed initial litter quality) mainly had a direct effect on litter mass loss in the unpolluted soils, while in polluted soils it changed to be an indirect effect. This denotes that the critical causality of PLE only occurs well in the polluted environment, where the lignin loss is also better explained (with a bigger *R* ^2^; Figure 6). Consequently, in an uncontaminated environment, due to diverse decomposer‐regulated function complexes, the lignin degradation became too complicated to be well explained. Meanwhile, it also had a greater explanatory power of litter decomposition speed for polluted incubation. In the unpolluted mesocosms, there was only a direct legacy effect of initial litter quality (caused by pre‐irradiation), with no significant indirect effect via lignin loss (absence of the primary causality path of PLE; Figure 6). Consequently, in MPs polluted soils, the offset effect of soil animals on microbial‐mediated PLE can be retarded, and the altered activities of animals and microbes can synthetically amplify the effect size of PLE, particularly on the biodegradation of lignin in the early phase of litter decomposition.

Together, although HDPE‐MPs could not significantly change litter mass loss, they might affect forest biogeochemical cycling by altering the performances of decomposer‐regulated PLE on marcescent litter decomposition. A better integration of anthropogenic chemical pollution in ecosystem function studies, such as litter decomposition, will further our understanding of how specific functions mediated and regulated by diverse organisms would alter under contamination. This is particularly crucial for the previously overlooked ecological processes, such as PLE in deciduous forests and subsequent implications for biogeochemical cycling.

## Author Contributions

**Kai Tian:** conceptualization (equal), formal analysis (equal), funding acquisition (equal), investigation (equal), methodology (equal), project administration (equal), resources (equal), software (equal), supervision (equal), writing – original draft (equal), writing – review and editing (equal). **Xin Wang:** conceptualization (equal), data curation (equal), formal analysis (equal), investigation (equal), methodology (equal), software (equal), visualization (equal), writing – original draft (equal), writing – review and editing (equal). **Rumeng Ye:** resources (equal). **Yingqi Wang:** investigation (equal). **Zhicheng Chen:** investigation (equal), writing – review and editing (equal). **Xiaojing Liu:** resources (equal). **Wenxia Wang:** resources (equal). **Lunguang Yao:** funding acquisition (equal), resources (equal), supervision (equal), writing – review and editing (equal).

## Conflicts of Interest

The authors declare no conflicts of interest.

## Supporting information


Data S1.

## Data Availability

Data S1 is available online at: https://doi.org/10.6084/m9.figshare.25524514.v3. Raw sequence data for microbial communities are available in the Sequence Read Archive (SRA) database of the National Centre for Biotechnology Information (NCBI) with accession number SRP487015. Data and code used in this study are available online from the Dryad Digital Repository: https://doi.org/10.5061/dryad.1g1jwsv4m (Reviewer URL: https://datadryad.org/stash/share/KeeGgTozOCspWIcjdVv0nT1YoDwDwX3AiLmMZ1Vk7k0).
