# Supplementary material for: Soil Decomposer Can Regulate the Legacy Effect of Photodegradation on Forest Marcescent Litter Decomposition, but Emerging Microplastics Disrupt This
Source: Ecol Evol. 2025 Jan 28;15(2):e70918. doi: 10.1002/ece3.70918 (PMC11775386; doi:10.1002/ece3.70918)
Supplement: Supplementary file 1 — Data S1. [file ECE3-15-e70918-s001.docx]

Supporting information for:

**Soil decomposer can regulate the legacy effect of photodegradation on forest marcescent litter decomposition, but emerging microplastics disrupt this**

Number of Pages: 17 (Contains: Tables, Figures and Biochemical assays procedures)

Number of Tables: 7

Number of Figures: 8

**Tables**

**Table S1.** Statistics of the PERMANOVA used to analyze the three binary variables coding effects of soil animals (A), HDPE microplastics (H) and photodegradation (P) on the enzyme activities. Significant P values (< 0.1) are highlighted in bold.

| **Source of variance** | ***df*** | **R²** | ***F*** | ***P*** |
| --- | --- | --- | --- | --- |
| **A (Soil animals)** | 1 | 0.189846 | 7.610745 | **0.001** |
| **H (HDPE microplastic)** | 1 | 0.063001 | 2.525657 | **0.067** |
| **P (Photodegradation)** | 1 | 0.011015 | 0.441568 | 0.68 |
| **A:H** | 1 | 0.081118 | 3.251957 | **0.04** |
| **A:P** | 1 | 0.019281 | 0.772948 | 0.469 |
| **H:P** | 1 | 0.005212 | 0.208924 | 0.823 |
| **A:H:P** | 1 | 0.031859 | 1.277206 | 0.295 |
|  | | | | |

**Table S2.** Pearson correlations (*ρ*) between the enzymatic traits (CBHI, cellobiohydrolase; BG, β-1,4-glucosidase; BX, β-1,4-xylosidase; URE, urease; NR, nitrate reductase; ACP, acid phosphatase; Perox, peroxidase; Pheno, phenol oxidase) and the first two components of PCA of the enzyme activities (PC1 and PC2). Significant P values (< 0.1) are highlighted in bold.

| **variable** | **PC1** | | **PC2** | |
| --- | --- | --- | --- | --- |
|  | ***ρ*** | ***P*** | ***ρ*** | ***P*** |
| **Pheno** | -0.866 | **0** | 0.163 | 0.374 |
| **Perox** | -0.935 | **0** | 0.122 | 0.507 |
| **Ure** | 0.274 | 0.129 | 0.579 | **0.001** |
| **NR** | 0.132 | 0.471 | 0.781 | **0** |
| **ACP** | -0.191 | 0.295 | -0.237 | 0.191 |
| **CBH1** | -0.636 | **0** | 0.454 | **0.009** |
| **BG** | 0.283 | 0.117 | 0.594 | **0** |
| **BX** | 0.302 | **0.094** | 0.225 | 0.216 |

**Table S3**. Effect of remaining dry weight, lignin content, total carbon, total nitrogen content, C:N ratio and lignin:N ratio of litter treated with and without abiotic ageing under UV radiation (t-test, *P* < 0.05, n = 4). Significant P values (< 0.05) are highlighted in bold.

| **variable** | ***t*** | ***df*** | ***P*** |
| --- | --- | --- | --- |
| mass remaining | -11.88 | 6 | **0.001** |
| lignin | -16.15 | 6 | **0.002** |
| Total C | -0.88 | 6 | 0.4116 |
| Total N | 6.95 | 6 | **0.001** |
| C:N | -6.19 | 6 | **0.001** |
| lignin:N | -9.8 | 6 | **0.001** |

**Table S4.** Difference of litter mass loss and chemical compositions (Closs, Nloss, lignin loss, C:N and lignin: N) during litter decomposition of *Lindera glauca* in eight treatments (mean ± SD, n=4). Significant differences (*P* < 0.05) are denoted by different letters.

| **Treatment (n=4)** | **mass loss (%)** | **C loss (%)** | **N loss (%)** | **lignin loss (%)** | **C: N** | **lignin: N** |
| --- | --- | --- | --- | --- | --- | --- |
| P-H-A- | 32.37±5.21ab | 39.11±6.76b | 18.84±18.84ab | 56.32±2.33c | 17.88±4.97ab | 13.02±2.4ab |
| P-H-A+ | 29.88±6.13ab | 37.51±3.77b | -0.49±38.63bc | 45.39±6.13abc | 24.8±10.55b | 21.11±8.17b |
| P-H+A- | 31.02±3.47ab | 36.22±7.56ab | -40.05±8.67a | 47.16±3.85abc | 15.48±2.57ab | 13.14±1.8ab |
| P-H+A+ | 27.15±5.53ab | 29.35±4.88a | -14.02±22.85ab | 32.04±6.59a | 20.77±5.09ab | 21.2±4.29b |
| P+H-A- | 36.84±2.96ab | 42.64±5.54b | 35.32±9.32c | 50.67±21.56c | 24.48±4.91b | 18.19±5.33ab |
| P+H-A+ | 30.15±6.15ab | 37.42±2.56b | 4.94±26.44bc | 37.95±13.29abc | 20.32±8ab | 17.65±8.36b |
| P+H+A- | 35.2±3.46ab | 41.76±3.53ab | -11.58±8.23ab | 53.64±17.20abc | 14.24±1.95a | 10.22±3.94ab |
| P+H+A+ | 32.45±1.75ab | 35.78±3.38a | 7.69±24.42ab | 54.2±5.5c | 19.25±5.57ab | 12.74±3.5ab |

**Table S5.** Statistics of the three-way ANOVA used to analyze the effects of litter photodegradation, HDPE microplastic and soil animals (with or without) on eight soil exoenzyme activities (CBH1, cellobiohydrolase; BG, β-1,4-glucosidase; BX, β-1,4-xylosidase; NR, nitrate reductase; URE, urease; ACP, acid phosphatase; Pheno, phenol oxidase; Perox, phenol oxidase). Significant or marginally significant relationships P- values (*P* < 0.1) are highlighted in bold.

| **Factors** | ***df*** | **CBH1** | | **BG** | | **BX** | | **Pheno** | | **Perox** | | **Ure** | | **ACP** | | **NR** | |
| --- | --- | --- | --- | --- | --- | --- | --- | --- | --- | --- | --- | --- | --- | --- | --- | --- | --- |
|  |  | ***F*** | ***P*** | ***F*** | ***P*** | ***F*** | ***P*** | ***F*** | ***P*** | ***F*** | ***P*** | ***F*** | ***P*** | ***F*** | ***P*** | ***F*** | ***P*** |
| **P (Photodegradation)** | 1 | 0.01 | 0.9 | 0.4 | 0.6 | 4.75 | **0.04** | 0.17 | 0.68 | 0 | 0.97 | 2.71 | 0.11 | 5.04 | **0.03** | 2.11 | 0.16 |
| **H （HDPE microplastics）** | 1 | 3.98 | ***0.1*** | 0.2 | 0.6 | 3.2 | ***0.09*** | 1.66 | 0.21 | 4.03 | ***0.06*** | 0.21 | 0.65 | 0.71 | 0.41 | 1.15 | 0.29 |
| **A (Soil fauna)** | 1 | 1.69 | 0.2 | 10 | **0** | 14 | **0** | 2.14 | 0.16 | 5.29 | **0.03** | 4.4 | **0.05** | 1.28 | 0.27 | 1.29 | 0.27 |
| **P : H** | 1 | 1.45 | 0.2 | 0.1 | 0.7 | 0.02 | 0.9 | 1.23 | 0.28 | 0.69 | 0.41 | 1 | 0.33 | 3.47 | ***0.1*** | 0.58 | 0.46 |
| **P : A** | 1 | 0.4 | 0.5 | 0.1 | 0.8 | 4.62 | **0.04** | 0 | 0.99 | 0.27 | 0.61 | 3.6 | ***0.07*** | 13.47 | **0** | 0.17 | 0.68 |
| **H : A** | 1 | 4.22 | ***0.1*** | 3.3 | ***0.1*** | 0.38 | 0.54 | 1.19 | 0.29 | 2.79 | 0.11 | 0.19 | 0.67 | 0.05 | 0.82 | 0.04 | 0.85 |
| **P : H : A** | 1 | 0.02 | 0.9 | 0.5 | 0.5 | 6.24 | **0.02** | 0.15 | 0.7 | 0.11 | 0.74 | 0.93 | 0.34 | 3.61 | ***0.1*** | 0.01 | 0.91 |

**Table S6**. Initial nutrient content of litter. Values are means ± SDs (n=4).

| Litter initial trait | Values |
| --- | --- |
| Organic carbon (%) | 46.61±0.95 |
| Total nitrogen (%) | 1.38±0.07 |
| Lignin content (%) | 47.75±0.50 |

**Table S7.** Difference of litter mass loss and chemical compositions (CBH1 and BG) during litter decomposition of *Lindera glauca* in eight treatments (mean ± SD, n=4). Significant differences (*P* < 0.05) are denoted by different letters.

| treatment | CBH1(μmol·h^-1^·g^-1^) | BG (μmol·h^-1^·g^-1^) |
| --- | --- | --- |
| P-H-A- | 0.43±0.1a | 1.11±0.19ab |
| P-H-A+ | 0.56±0.08a | 0.89±0.17a |
| P-H+A- | 0.42±0.08a | 1.06±0.16ab |
| P+H-A- | 0.45±0.06a | 1.1±0.22ab |
| P+H-A+ | 0.54±0.11a | 0.89±0.05a |
| P+H+A- | 0.41±0.11a | 1.23±0.09b |
| P+H+A+ | 0.4±0.11a | 1.04±0.21ab |

**Figures**


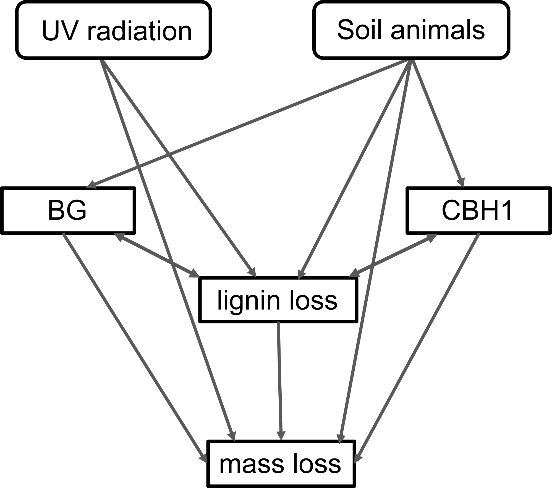


**Figure S1.** Conceptual structural equation model (SEM) summarizing *a priori* causal pathways in which two exogenous explanatory variables UV radiation and soil animals (both are binary variables), and three endogenous explanatory variables soil microbial enzymatic activities (CBH1 and BG), and lignin loss may influence litter mass loss. Single-headed arrows signify a hypothesized causal influence of one variable upon another. Double-headed arrows indicate a correlation in which no direction is specified. This *a priori* model was separately conducted for the with or without HDPE-MPs polluted decomposition systems.

**
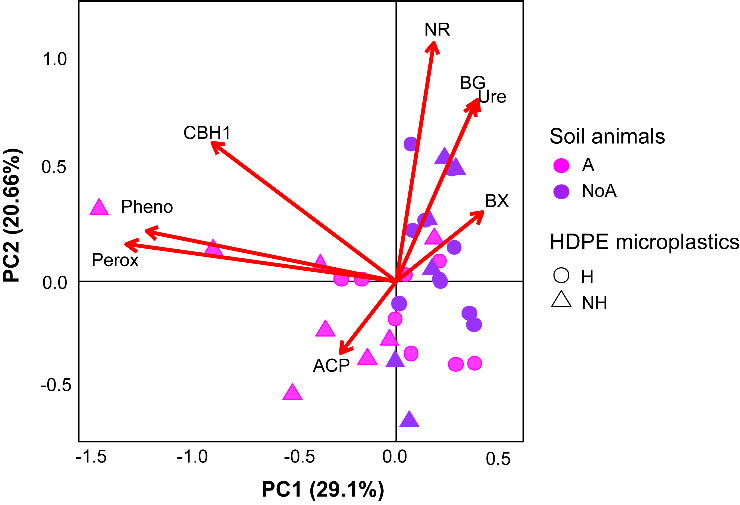
**

**Figure S2.** Principal component analysis (PCA) of eight soil ectoenzyme activities (CBH1, cellobiohydrolase; BG, β-glucosidase; BX, β-xylosidase; NR, nitrate reductase; URE, urease; ACP, acid phosphatase; Pheno, phenol oxidase; Perox, phenol oxidase), and interactive effects of soil animals and HDPE MPs (with or without) on enzymatic activities. Variances explained by the first two components (PC1 and PC2) are shown.

**
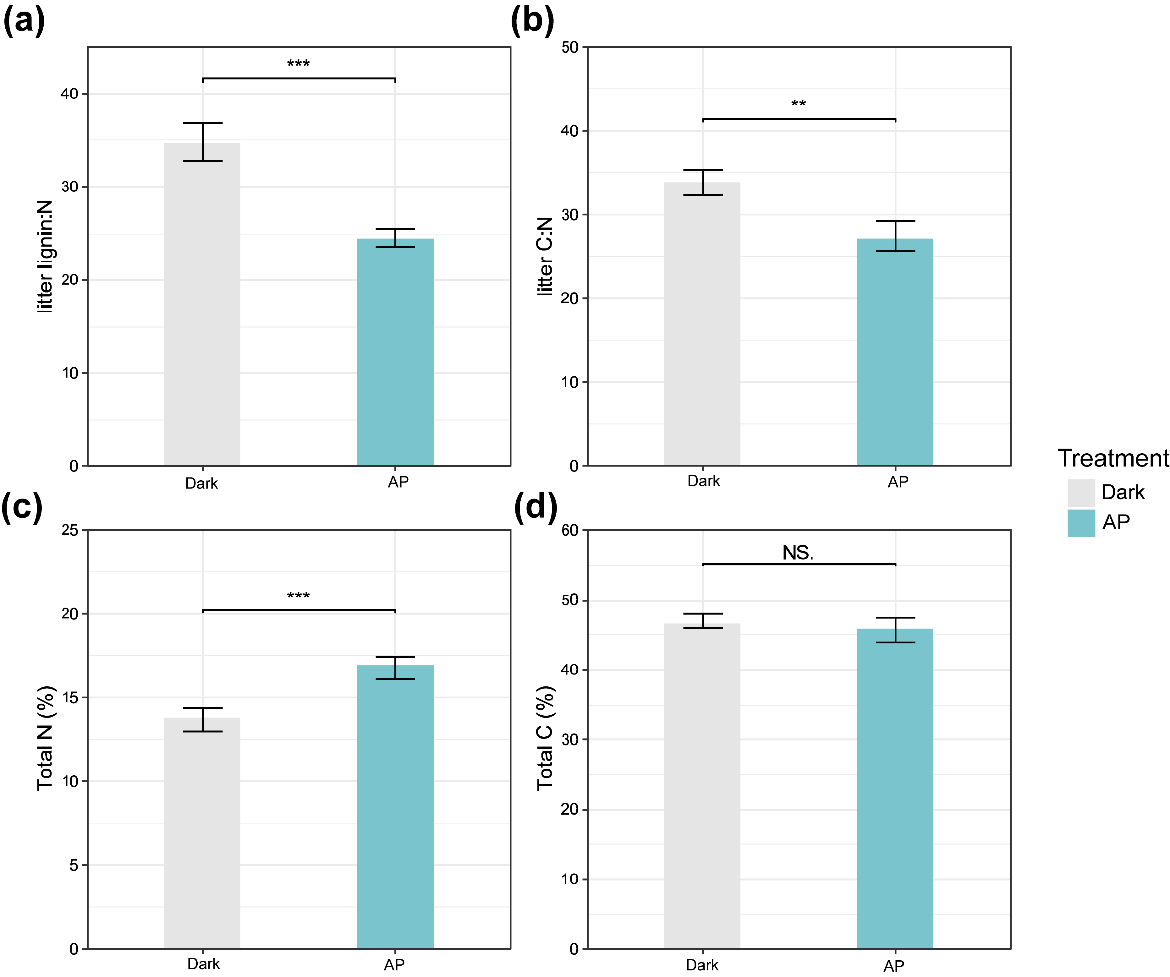
**

**Figure S3.** Different influences of abiotic ageing (AP, simulates marcescent photodegradation process, treated in the UV accelerated weathering tester box) and darkness (Dark, treated in sealed dark box) on (a) lignin: N, (b) C: N, (c) total N and (d) total C of freshly senescent *L. glauca* litters. Values are means ± SD (n = 4). Asterisks indicate significant difference between groups (**P* < 0.05, **P* < 0.01, ****P* < 0.001).

**
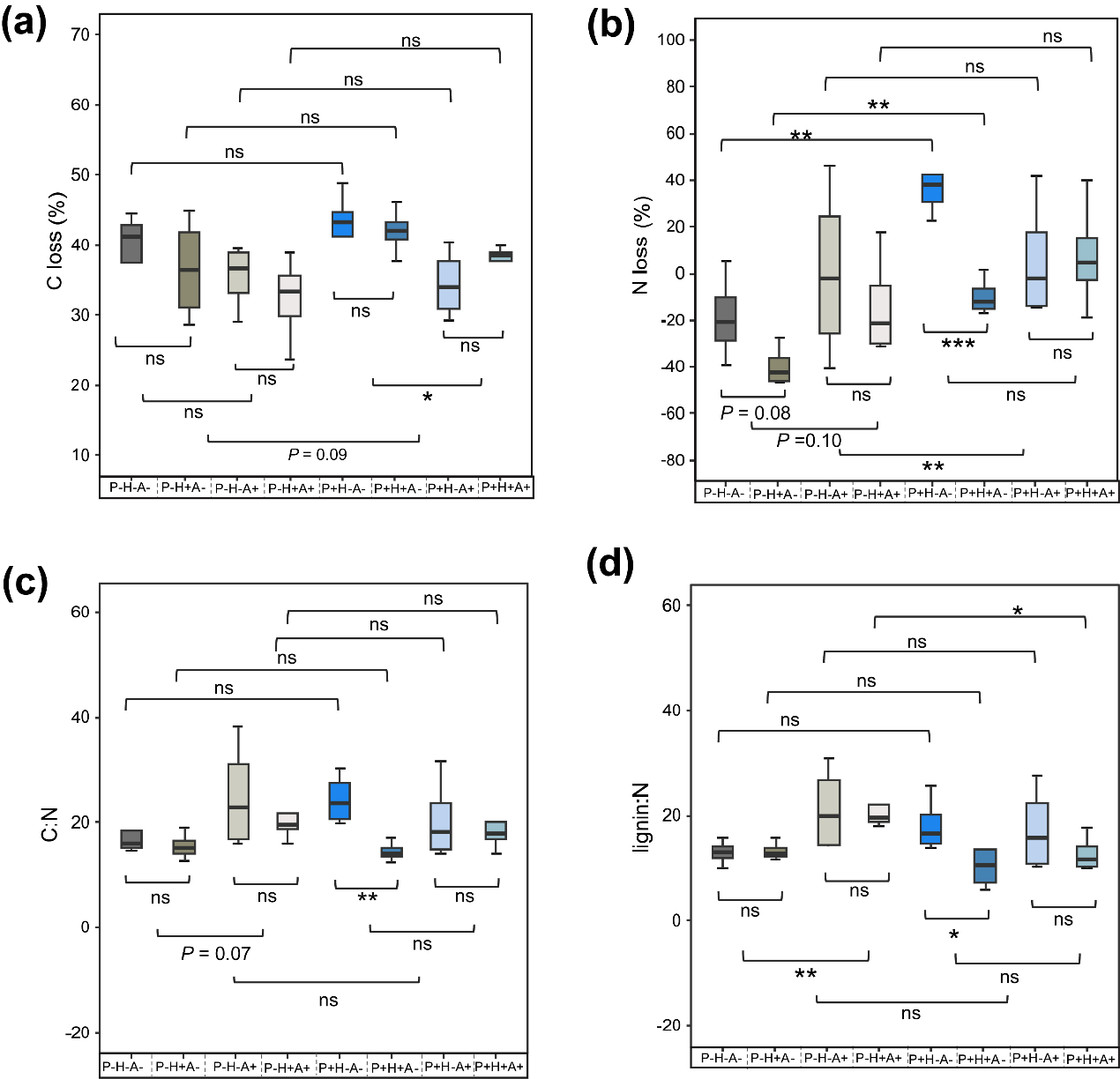
**

**Figure S4.** Interactive influences of photodegradation, soil animal and HDPE-MPs on litter (a) Closs, (b) N loss, (c) C:N and (d) lignin: N after 90 days incubation. Different colors represent various treatments involving P+H-A-, with photodegradation; P-H-A-, non-photodegradation; P+H+A-, photodegradation & HDPE; P-H+A-, non-photodegradation & HDPE; P+H-A, photodegradation & soil animals; P-H-A+, non-photodegradation & soil animals; P+H+A+, photodegradation & HDPE & Soil animals), P-H+A+, non-photodegradation & HDPE & soil animals. Asterisks indicate significant difference between groups (**P* < 0.05, **P* < 0.01, ****P* < 0.001).

**
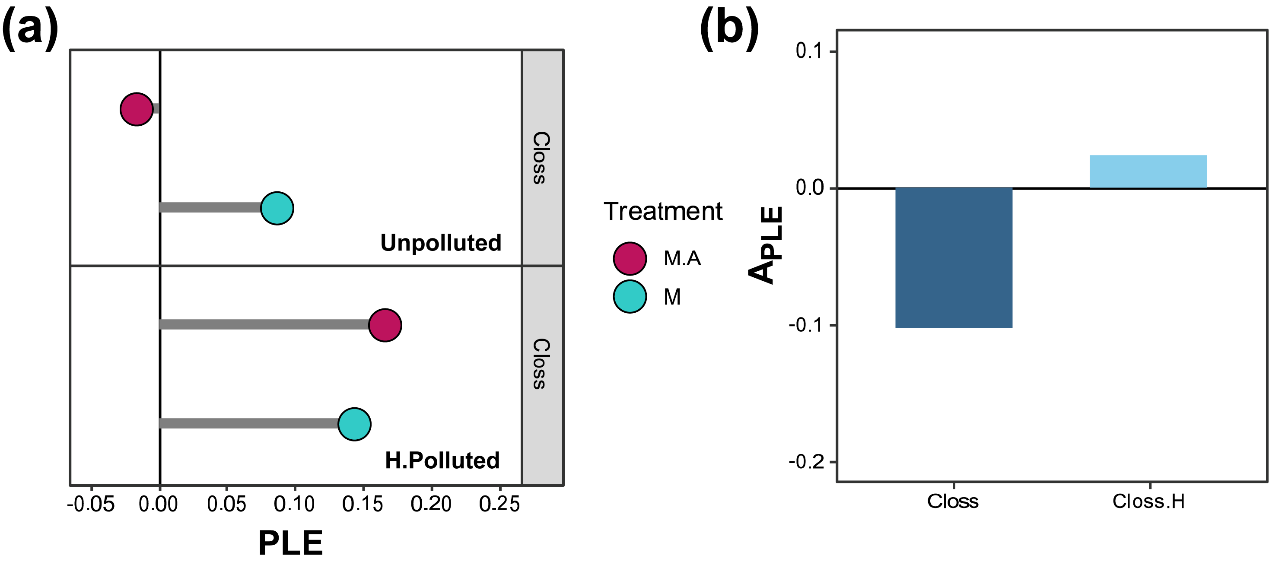
**

**Figure S5.** Effect size of the legacy effect of pre-photodegradation (PLE) on subsequent bio-decomposition. (a) In two contrasting environments that containing HDPE-MPs (H. Polluted) and not (Unpolluted), and with (M.A) or without (M) the impact of soil animals, the PLEs on Closs (*PLE_Closs_*) was shown. (b) Soil animal’s regulatory influences on PLE (*A_PLE_*) of Closs, H denoted incubated in soils contaminated by HDPE-MPs.


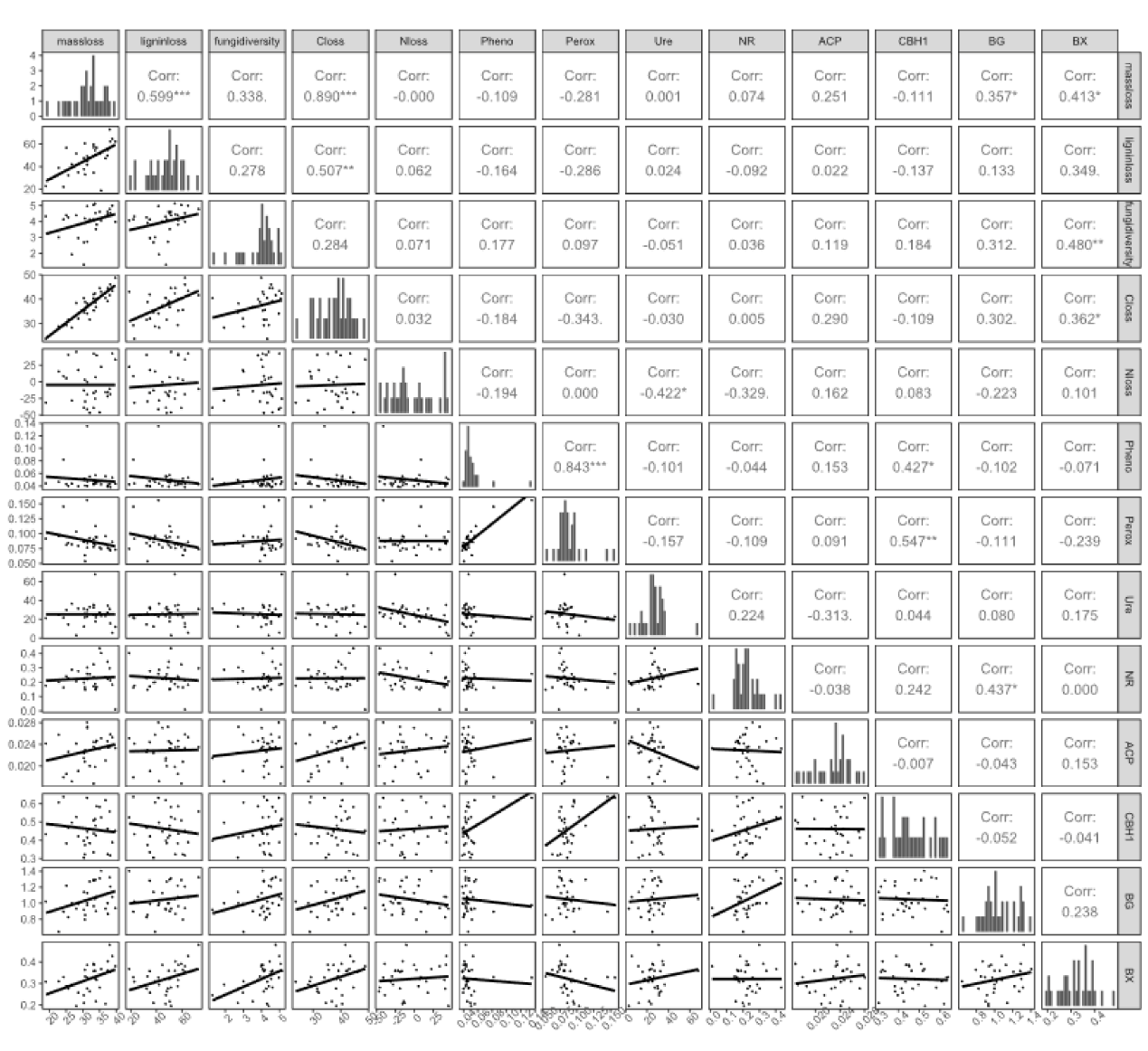


**Figure S6.** Multivariate regression analysis to analyze the correlation between litter mass loss, lignin loss, fungi diversity (H) and eight soil exoenzyme activities (soil carbon, nitrogen, and phosphorus cycling). The upper-right section displays Pearson correlation coefficients between variables, and asterisks indicate significant difference between variables (“***” *P* < 0.001; “**” *P* < 0.01; “*” *P* < 0.05). The lower-left section showcases linear relationships between variables.

**
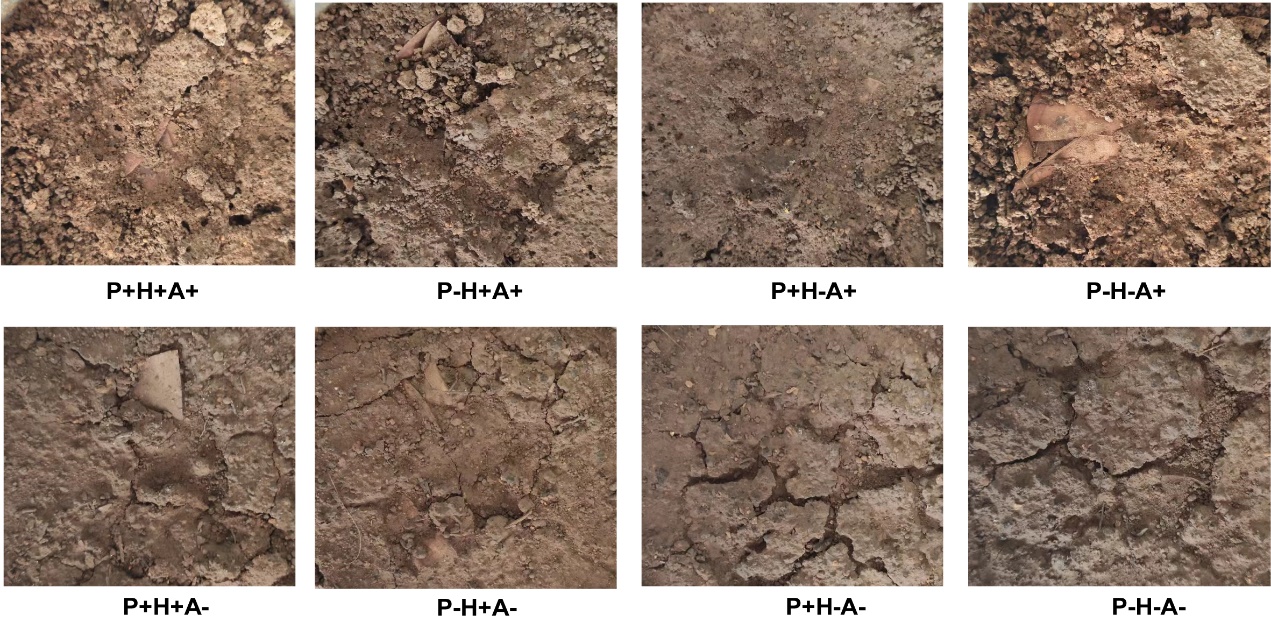
**

**Figure S7.** Comparation of incubation soils after a 90 days incubation. Surface soils were shown in each mesocosm, wherein vermicomposts spread everywhere in the animal added treatments (the above 4 ones). Experimental treatments: with Photodegradation (P+H-A-); No-photodegradation (P-H-A-); Photodegradation & HDPE-MPs (P+H+A-); No-photodegradation & HDPE-MPs (P-H+A-); Photodegradation & Soil animals (P+H-A+); No-photodegradation & Soil animals (P-H-A+); Photodegradation & HDPE-MPs & Soil animals (P+H+A+); No-photodegradation & HDPE -MPs & Soil animals (P-H+A+).

**
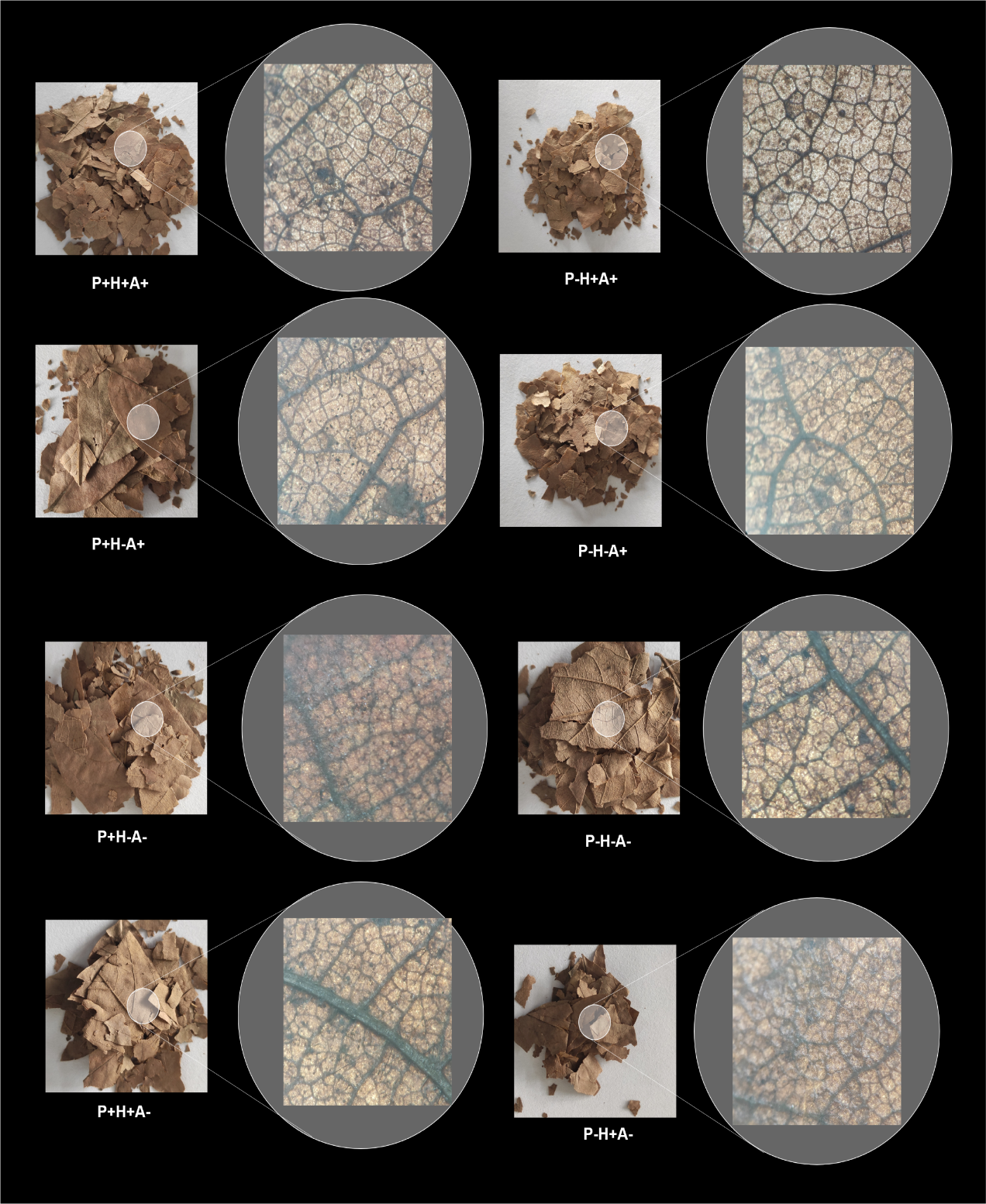
**

**Figure S8.** Litters that have been washed and air dried after 90 days incubation, showing mechanical fragmentation status. Experimental treatments: with Photodegradation (P+H-A-); No-photodegradation (P-H-A-); Photodegradation & HDPE-MPs (P+H+A-); No-photodegradation & HDPE-MPs (P-H+A-); Photodegradation & Soil animals (P+H-A+); No-photodegradation & Soil animals (P-H-A+); Photodegradation & HDPE-MPs & Soil animals (P+H+A+); No-photodegradation & HDPE -MPs & Soil animals (P-H+A+).


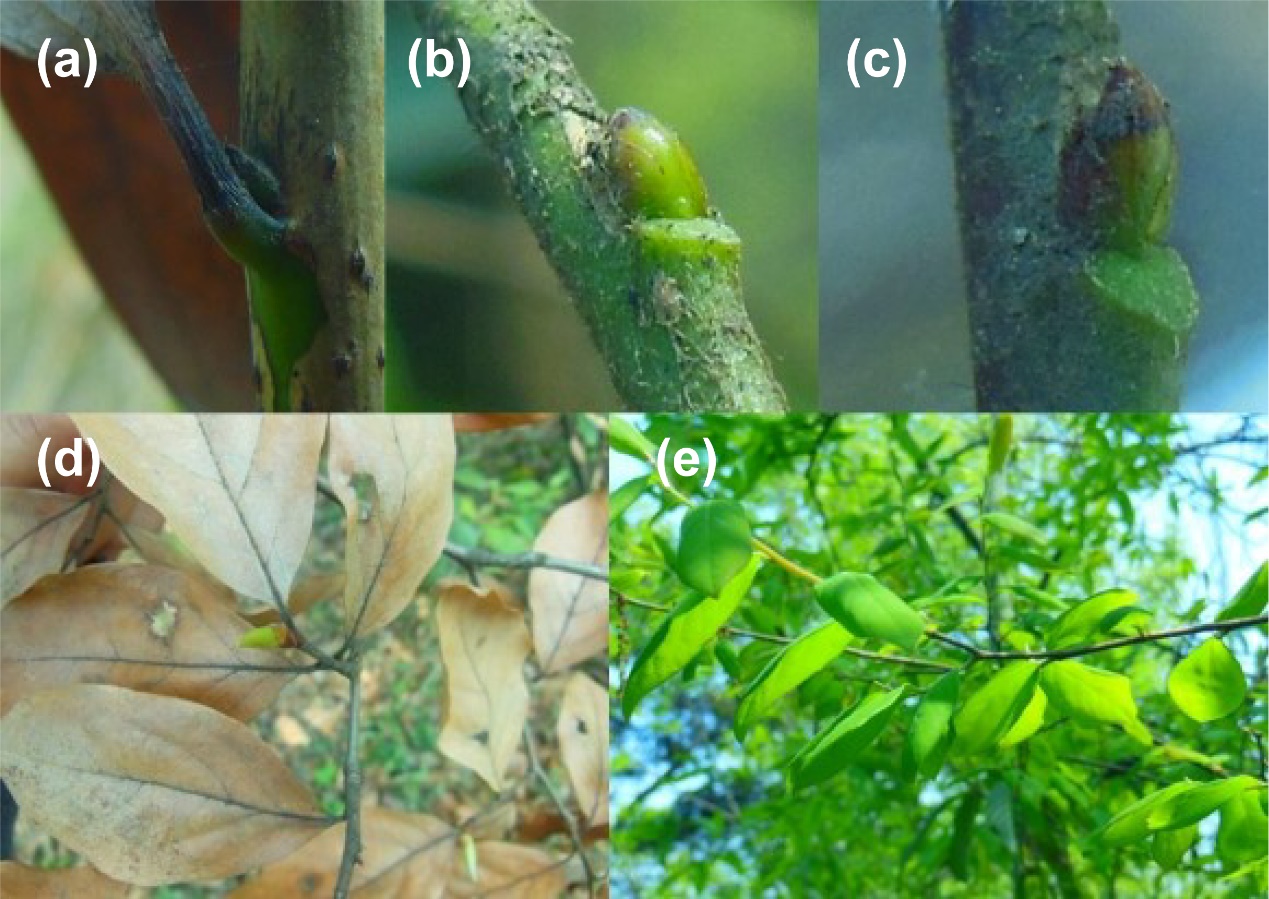


**Figure S9.** Photographs illustrating the petiole base structures of marcescent litter that support dead leaves hanging on until new leaves spread out in next spring. (a ~ c) *L. glauca* cellular lysis of abscission layer zone of its withered leaf is incompletely developed throughout the winter, (d ~ e) the marcescent leaves hanging on before they shed and fall into soil in spring.

**Biochemical assays:**

**Soil exoenzyme activities, microbial biomass carbon and nitrogen**

(1) Exoenzyme activity measurement:

Activities of cellobiohydrolase, β-1,4-glucosidase, and β-1,4-xylosidase were determined using 1.2 mM 4-nitrophenyl-β-d-linked (PNPX) substrates (cellobioside, glucopyranoside, xylopyranoside) with incubation in the dark at 40 °C for 1.5 h (pH 5.0, 0.2 M Na_2_CO_3_ was used to stop the reaction), 4-Nitrophenyl (PNP) concentrations were quantified by measuring absorbance at 400 nm using a microplate spectrophotometer (Tecan Safire2, Switzerland) in 96-well plates (Vepsalainen et al. 2001) (Vepsalainen et al. 2001). Enzymatic activities are expressed in μ mol PNP h^-1^ g^-1^ soil.

Phenol oxidase activities were measured spectrophotometrically using 100μL of 25 mM l-3,4-dihydroxyphenylalanine (L-DOPA) as the substrate with incubation at 26.5 °C for 1 h (pH 5.5; quantified by measuring absorbance at 450 nm using the microplate spectrophotometer in 96-well plates) (Saiya-Cork et al. 2002). Enzymatic activity are expressed in μ mol L-DOPA h^-1^ g^-1^ soil.

Phenol peroxidase activities were measured spectrophotometrically using 100 μL of 25 mM l-3,4-dihydroxyphenylalanine (L-DOPA) as the substrate with incubation at 26.5 °C for 1 h, then add 0.3% hydrogen peroxide (pH 5.5, quantified by measuring absorbance at 450 nm using the microplate spectrophotometer in 96-well plates) (Saiya-Cork et al. 2002). Enzymatic activity are expressed in μ mol L-DOPA h^-1^ g^-1^ soil.

Nitrate reductase activity was determined using 200 mM KNO_3_ solution as substrate with incubation at room temperature for 30 min (pH 7.5). NO^2−^ concentration was determined with a spectrophotometer (JingHua, Shanghai, China) at a wavelength of 520 nm (Daniel and Curran 1981). Enzymatic activity was quantified by reference to a calibration curve incubated with soil under the same conditions described and is expressed in μg NO^2−^ min^-1^ g^-1^ soil (Daniel and Curran 1981).

Urease activity was determined with urea as substrate, incubated at pH 6.7 (0.2 M phosphate buffer) and 37 °C for 24 h, and absorbance was measured at 578 nm using the spectrophotometer (Nannipieri et al. 1980). Enzymatic activity is expressed in mg NH_3_-N h^-1^ g^-1^ soil.

Acid phosphatase activities were determined using 0.5% disodium phenyl phosphate solution as substrate with incubation at 37 °C for 24 h (pH 5.0 for acid phosphatase), phenol concentration was determined with the spectrophotometer at 570 nm) (Kandeler et al. 1999). Enzymatic activities were quantified by reference to a calibration curve incubated with soil under the same conditions described and is expressed in mg phenol h^-1^ g^-1^ soil (Kandeler et al. 1999).

(2) Microbial biomass carbon (MBC) and nitrogen (MBN) measurement:

Soil MBC and MBN was estimated by the chloroform-fumigation-extraction method described. Concretely, 50 ml chloroform (without ethanol) and dilute NaOH (with zeolite) are placed in the vacuum dryer, then, weighed fresh soil (equivalent to 10g of dry soil) to 25 ml beaker. After vacuum pumping, soil was cultured in dark condition for 24h. At the same time, weighed equal parts of soil and treated as control without fumigation. Then, 0.5M potassium sulfate extraction (1:4), shaked culture and filtration by 0.2 μm filter, respectively. The concentration of MBC was measured by a K_2_Cr_2_O_7_- FeSO_4_·7H_2_O method. Concretely, 5ml extract liquor was placed into 50ml erlenmeyer flask, added 10ml 0.018M K_2_Cr_2_O_7_-H_2_SO_4_, and placed it on 200℃ electric stove, and boiled for 10 minutes. Added 80 ml distilled water and 30μl phenanthroline indicator, then 0.05M FeSO_4_ titration (orange-yellow to blue-green to brownish-red color change). Use silicon dioxide as a blank sample. MBC was calculated as follows: *MBC* = *E_C_* / *k_EC_*, where *E_C_* = (organic C extracted from fumigated soil) – (organic C extracted from non-fumigated soil) and *k_EC_* = 0.45, which is the proportionality factor to convert *E_C_* to MBC. The concentration of MBN was measured by a ninhydrin colorimetry method. Concretely, 0.5 ml sample extract liquor was placed into 10ml centrifuge tube, and added 2ml ninhydrin ethanolic solution, vortex mixing, then boiled water bath for 15 minutes, iced bath quickly after removal for 2min. Added 5ml ethanol solution, and then measured at 570 nm using the spectrophotometer.

**Litter lignin, total C and N content**

(1) Klason lignin:

Weighed 0.5g of air-dried litter powder (*W_1_*) and transfer it to a triangular flask, added 20ml of 72% sulfuric acid, stirring with a glass rod until a homogeneous mixture is achieved. Allowed the blend to stand at room temperature for 4 hours. Subsequently, introduce approximately 800ml of distilled water and reflux the mixture at high temperature on an electric stove for 2 hours. Utilize a constant-weight sand core crucible (*W_2_*) for filtration, followed by drying at 105°C until a constant weight is attained. Remove and weigh the crucible (*W_3_*). Calculate the lignin content using the formula: lignin% = (*W_3_*-*W_2_*) / *W_1_* × 100%. Repeat the procedure four times for enhanced precision.

(2) Litter total C:

Litter total carbon was measured potassium bichromate sur-hot process using 0.2g air-dried litter powder and placed it into 50ml erlenmeyer flask, added 10ml 0.4M K_2_Cr_2_O_7_-H_2_SO_4_, and soak for 24~48 hours. Then, place it on electric stove, and boiled for 5 minutes. Added 50 ml distilled water and 30~60 μl phenanthroline indicator, then 0.2M FeSO_4_ titration (orange-yellow to blue-green to brownish-red color change). Use silicon dioxide as a blank sample.

(3) Litter total N:

Litter total nitrogen was measured indophenol blue colorimetry using 0.5g air-dried litter powder and placed it into bottom of a conical flask (GG-17), soaked 5ml sulfuric acid overnight. Then, place it on electric stove (250 ℃), when white smoke disappears, added 60% perchloric acid in batches (100μl each time), until the sample turns white. Cool the conical flask, added distilled water and set to 100ml.

Pipette 2 mL or 3 mL of the tenfold ashing into a 25 mL colorimetric tube, added 1 mL of methyl red-EDTA, 1M NaOH (pH to 6), 2.5ml Phenol solution and 2.5ml sodium hypochlorite solution, respectively, and constant volume. After 1h, quantified by measuring absorbance at 625 nm using the microplate spectrophotometer in 96-well plates.

**References:**

Daniel, R. M., and M. P. Curran. 1981. A Method for the Determination of Nitrate Reductase. Journal of Biochemical and Biophysical Methods 4:131-132. <https://doi.org/10.1016/0165-022X(81)90026-9>

Kandeler, E., D. Tscherko, and H. Spiegel. 1999. Long-term monitoring of microbial biomass, N mineralisation and enzyme activities of a Chernozem under different tillage management. Biology and Fertility of Soils 28:343-351. <https://doi.org/10.1007/s003740050502>

Love, M. I., Huber, W., & Anders, S. (2014). Moderated estimation of fold change and dispersion for rna-seq data with deseq2. Genome Biology, 15(12), 550. <https://doi.org/10.1186/s13059-014-0550-8>

Mandal, S. , Van Treuren, W. , White, R. A. , Eggesb?, M. , Knight, R. , & Peddada, S. D. . (2015). Analysis of composition of microbiomes: a novel method for studying microbial composition. Microbial Ecology in Health & Disease, 26. <http://dx.doi.org/10.3402/mehd.v26.27663>

Nannipieri, P., B. Ceccanti, S. Cervelli, and E. Matarese. 1980. Extraction of Phosphatase, Urease, Proteases, Organic-Carbon, and Nitrogen from Soil. Soil Science Society of America Journal 44:1011-1016. <https://doi.org/10.2136/sssaj1980.03615995004400050028x>

Saiya-Cork, K. R., R. L. Sinsabaugh, and D. R. Zak. 2002. The effects of long term nitrogen deposition on extracellular enzyme activity in an Acer saccharum forest soil. Soil Biology & Biochemistry 34:1309-1315. <https://doi.org/10.1016/S0038-0717(02)00074-3>

Segata, N., Izard, J., Waldron, L. et al. Metagenomic biomarker discovery and explanation. Genome Biol 12, R60 (2011). <https://doi.org/10.1186/gb-2011-12-6-r60>

Vepsalainen, M., S. Kukkonen, M. Vestberg, H. Sirvio, and R. M. Niemi. 2001. Application of soil enzyme activity test kit in a field experiment. Soil Biology & Biochemistry 33:1665-1672. https://doi.org/10.1016/S0038-0717(01)00087-6
